# Influence of commensal bacteria on the proteolytic and antigenic profiles of INFOGEST-like digested wheat gliadin

**DOI:** 10.3389/fmicb.2026.1842801

**Published:** 2026-07-06

**Authors:** Flávio Pereira-Costa, Vanessa S. Domingues, Ana Roque, Zaida L. Almeida, Pedro F. Cruz, Rachel Cordeiro, Daniela Trindade, Carla Moura, Joana B. Melo, Sónia G. Pereira, Daniela C. Vaz

**Affiliations:** 1 CiTechCare, Centre for Innovative Care and Health Technology, School of Health Sciences, Polytechnic Institute of Leiria, Leiria, Portugal; 2 Department of Life Sciences, NOVA School of Science and Technology, Universidade NOVA de Lisboa, Caparica, Portugal; 3 Department of Chemistry, Coimbra Chemistry Centre (CQC), Institute of Molecular Sciences (IMS), University of Coimbra, Coimbra, Portugal; 4 School of Health Sciences, Polytechnic Institute of Leiria, Leiria, Portugal; 5 CDRsp, Centre for Rapid and Sustainable Product Development, Polytechnic of Leiria, Marinha Grande, Portugal; 6 Veterinary Clinics Department, Abel Salazar Biomedical Sciences Institute (ICBAS), University of Porto, Porto, Portugal; 7 Polytechnic University of Coimbra, Coimbra, Portugal; 8 Research Centre for Natural Resources, Environment and Society (CERNAS), Polytechnic University of Coimbra, Coimbra, Portugal; 9 Cytogenetics and Genomics Laboratory and iCBR-CIMAGO, Faculty of Medicine, University of Coimbra, Coimbra, Portugal; 10 Associate Laboratory LSRE-LCM, Laboratory of Separation and Reaction Engineering and Laboratory of Catalysis and Materials, Polytechnic Institute of Leiria, Leiria, Portugal

**Keywords:** alpha-gliadin, antigenic gliadin fragments, celiac disease, gliadin-degrading bacteria, INFOGEST *in vitro* digestion, protein aggregates, proteolytic cleavage, anti-gliadin ELISA

## Abstract

**Introduction:**

Celiac disease (CeD) is a chronic autoimmune enteropathy developed by genetically predisposed individuals when exposed to gluten. Gluten gliadins, along with gut microbiota, may influence CeD onset and progression through mechanisms that remain unclear.

**Methods:**

Gliadin-degrading bacterial isolates obtained from CeD patients, and their 1st-degree relatives’ stool and blood were identified (*Bacillus tropicus*, *Enterococcus faecalis*, *Micrococcus* sp., *Cronobacter sakazakii*, *Pseudomonas aeruginosa*, and *Serratia marcescens*) and used in an INFOGEST-like protocol to simulate gliadin digestion after 4 h (digested gliadin, d-gliadin). The d-gliadin digesta were analyzed by fast protein liquid chromatography (FPLC), dynamic light scattering (DLS), Fourier transform infrared spectroscopy (FTIR), scanning electron microscopy (SEM), fluorescence spectroscopy, and polyclonal and monoclonal (R5 and G12) enzyme-linked immunosorbent assays (ELISA).

**Results and discussion:**

In the absence of the bacterial isolates, gliadin is poorly digested and self-assembles within 1 day into intermediate and large protein oligomers/aggregates, enriched in β-sheet structure (FTIR amide I band between 1,600 and 1,700 cm^−1^) and able to bind thioflavin T and Congo red. Conversely, in the presence of the bacterial isolates, gliadin is further digested, leading to an increase in protein fragments. After 4 h, the *P. aeruginosa*, *C. sakazakii*, and *B. tropicus* d-gliadin digesta presented a mixture of d-gliadin peptides and aggregates that showed higher antigenicity (associated with the exposure of the 5-amino acid QQPFP and 6-amino acid QPQLPY epitopes, present in the 25-mer and 33-mer, respectively) than control digestions (without bacteria), while *E. faecalis* led to lower antigenicity. In turn, within 24 h of incubation, all bacterial isolates led to the formation of undigested material with lower antigenicity, either due to fewer 33-mers and 25-mers in solution, or to fragment aggregation into amorphous material, not exposing antigenic sequences. Hence, intestinal flora may enhance or diminish the antigenicity of gliadin, thereby modulating the immunogenic response to gliadin/gluten.

## Graphical abstract

**Figure:**
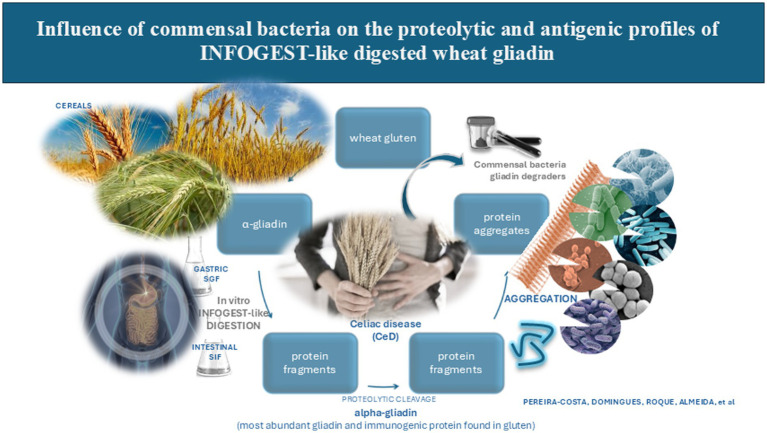

## 1 Introduction

Celiac disease (CeD) is a prevalent autoimmune disorder that affects approximately 1–2% of the worldwide population (Catassi et al., 2022). Although genetic predisposition is required, with human leukocyte antigen (HLA)-DQ2 and/or HLA-DQ8 genes defined as CeD-related genes, only 2–3% of gene carriers develop the disease (Leonard and Vasagar, 2014). Another determinant of the disease is its trigger, dietary gluten, a complex mixture of grain storage proteins found in wheat, barley, and rye. In individuals with CeD, exposure to gluten leads to an immune response, characterized by the production of antibodies against deamidated gliadin and autoantibodies against tissue transglutaminase-2 (TG2), resulting in intestinal damage and in a range of systemic symptoms (Ludvigsson et al., 2013). Indeed, while being the only autoimmune disease with a known trilogy, of genetic predisposition, environmental trigger, and auto-antibodies, it is not yet understood: (a) why only a small part of the genetic carriers develops CeD? (b) why some genetic carriers with high titers of anti-TG2 auto-antibodies remain asymptomatic? and (c) why others with a strict gluten-free diet remain symptomatic? (Raiteri et al., 2022) Thus, exploring other disease-related features might deliver valuable insights to understand CeD.

Wheat gluten is mainly constituted by two fractions: glutenins and gliadins. Gliadins are monomeric proteins classified in four groups (ω5; ω1, 2; α; and γ) based on their electrophoretic mobility (Caldwell, 1983). The molecular weights of gliadins range from 20 to 55 kDa, being alpha (α) gliadins (20 to 35 kDa), the most abundant proteins found in gluten (Wieser et al., 2023). Moreover, it is well established that alpha-gliadins are the most immunogenic gluten proteins in CeD (Stănciuc et al., 2018). The elevated glutamine (Q) and proline (P) content in gliadin makes the protein mostly resistant to gastrointestinal digestion enzymes (Shan et al., 2005). Alpha-gliadin peptides (Mamone et al., 2007), such as the 33-mer, 25-mer, and 13-mer, remain undigested and can translocate the intestinal barrier (Cebolla et al., 2018), inducing inflammation/immune responses, as previously observed in animal and cell models (Barone et al., 2014; Gómez Castro et al., 2019; Herrera et al., 2018; Nanayakkara et al., 2018).

In addition, environmental factors, such as the intestinal microbiota, which influence nutrient absorption, barrier function, and mucosal immune maturation, have been proposed to be relevant to the onset and progression of CeD (Rossi et al., 2023). Some studies have shown that certain bacteria are capable of degrading gliadin peptides resistant to human gastrointestinal enzymes (Caminero et al., 2016; Sánchez et al., 2012; Caminero et al., 2019; Laparra and Sanz, 2010). Additionally, intestinal microbiota dysbiosis has been identified as a risk factor for CeD, and an increase in Gram-negative bacteria compared to Gram-positive species has been reported in CeD patients (Girbovan et al., 2017). Hence, even if these studies have highlighted the possible role of specific bacterial strains in increasing or reducing the immunogenic properties of gliadin/gluten, it remains unknown whether this could be related to the onset and development of CeD.

Hence, the purpose of our study was to investigate the ability of commensal bacterial isolates obtained from fecal and blood samples of CeD patients and their non-celiac 1st-degree relatives to degrade gliadin *in vitro* and to further characterize the digesta. *In vitro* digestions were carried out by making use of the standardized INFOGEST 2.0 protocol (Brodkorb et al., 2019), which has already been applied to study the digestibility of various types of food (e.g., beverages, vegetables, and meat), nutrients (e.g., lipids and proteins), and bioactive compounds (e.g., polyphenols and carotenoids) (Blanco-Morales et al., 2018; Ferrara et al., 2025; Lavoisier et al., 2023; Llorens et al., 2024; Lucas-González et al., 2023; Nehir El et al., 2015; Spréa et al., 2026; Tenenbaum et al., 2023; Xiao et al., 2026). To accomplish this, we have: (I) cultured blood and stool bacterial isolates in gliadin-rich agar plates to identify gliadin-degraders; (II) conducted *in vitro* static digestions in the absence and presence of the selected bacteria; (III) analyzed the protein entities produced by size-exclusion fast protein liquid chromatography (FPLC), scanning electron microscopy (SEM), Congo-Red and Thioflavin-T (ThT) binding, dynamic light scattering (DLS), and Fourier-transform infrared spectroscopy (FTIR); and (IV) evaluated the antigenic potential of the digesta by enzyme-linked immunosorbent assays (ELISAs) against polyclonal and monoclonal R5 and G12 antibodies.

## 2 Experimental section

### 2.1 Culture of stool and blood samples

Stool (*n* = 133) and blood (*n* = 116) bacterial isolates, previously obtained from CeD patients and their non-CeD first-degree relatives (Roque et al., 2024), were used to isolate CeD-related commensal bacteria. Blood samples (500 μL) were cultured in duplicates in Brain Heart Infusion (BHI) broth aerobically at 37 °C, 150 rpm, for 5 days and then sub-cultured onto sheep blood, chocolate, and MacConkey agar media at 37 °C for 72 h. Direct culturing (500 μL) on agar plates was also performed with the same whole blood samples, growth media, and conditions. Stool samples (~10 μg) were cultured on Man–Rogosa–Sharpe (MRS) and MacConkey agar at 37 °C, from 24 h to 72 h, under aerobic conditions.

### 2.2 Screening of CeD-related commensal gliadin-degrading bacteria

**Figure 1:**
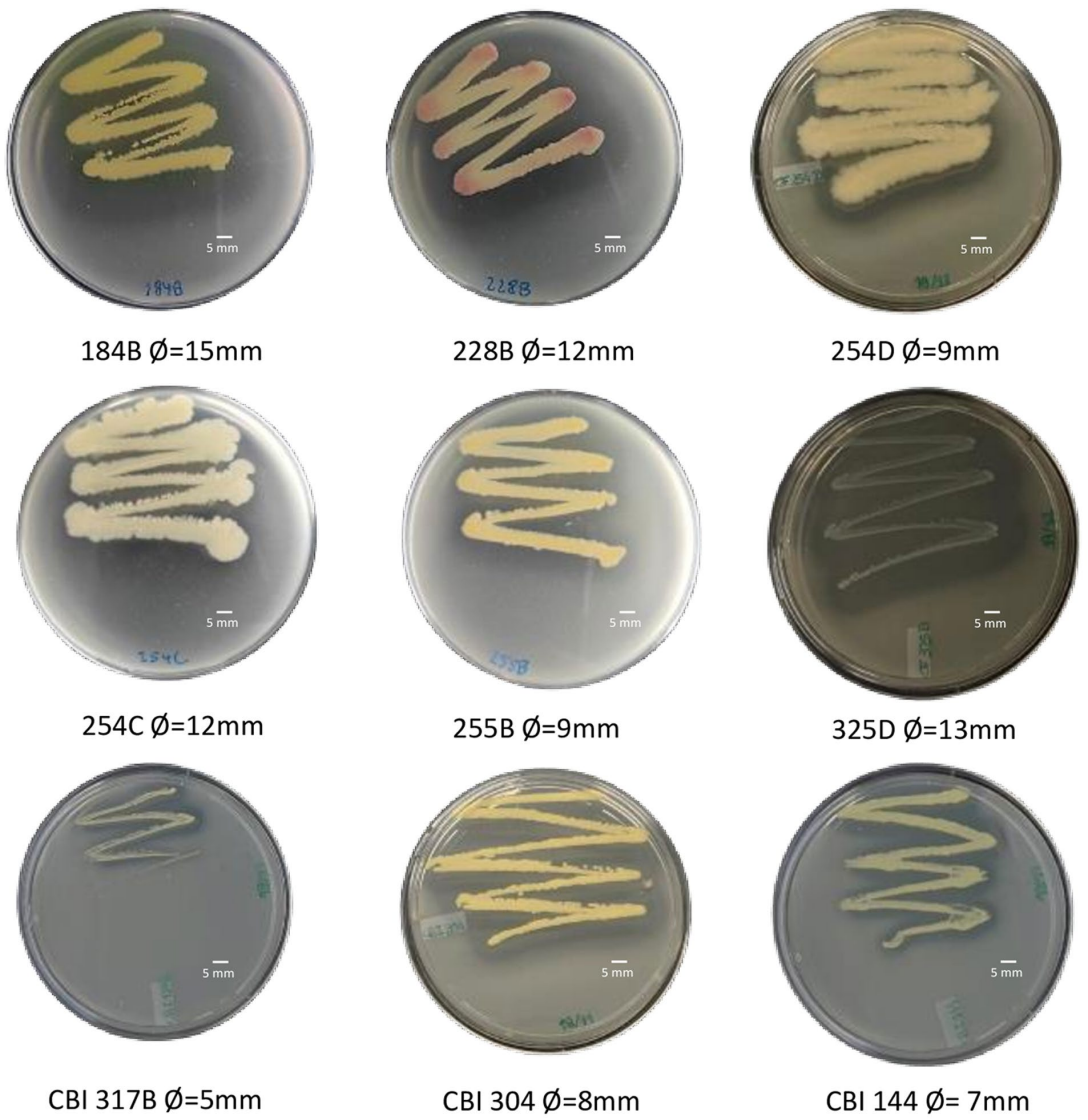
Agar plates of gliadin-degrading bacterial isolates identified by 16S rRNA gene sequencing: 184B, *Pseudomonas aeruginosa* (Ø 15 mm); 228B, *Serratia marcescens* (Ø 12 mm); 254D, *Bacillus tropicus* (Ø 9 mm); 254C, *Bacillus tropicus* (Ø 12 mm); 255B, *Cronobacter sakazakii* (Ø 9 mm); 325D, *Enterococcus faecalis* (Ø 13 mm); CBI317B, *Micrococcus luteus* (Ø 5 mm); CBI304; *Micrococcus luteus* (Ø 8 mm) and CBI144, *Micrococcus* sp. (Ø 7 mm). The halo/clear zone around the bacteria (≥5 mm) demonstrates gluten-degrading activity on the agar plates supplemented with 0.2% (w/v) wheat gliadin.

Randomly selected stool and blood bacterial isolates were screened for gliadin-degrading activity according to Berger et al. (2015). Briefly, bacteria were cultured in tryptic soy agar (TSA) medium supplemented with 0.2% (w/v) wheat gliadin (Sigma-Aldrich, Germany) at 37 °C for 72 h under aerobic conditions (Supplementary Figure S1). A clear halo around the bacterial colonies was considered indicative of gliadin degradation. Bacterial isolates with a halo ≥ 5 mm were selected for further identification (Figure 1) and were added to gliadin in the intestinal phase (SIF) of an *in vitro* static INFOGEST-like digestion protocol of gliadin.

### 2.3 Bacterial identification by 16S rRNA gene sequencing

DNA was extracted from bacterial colonies using the boiling method (Sepp et al., 1994). The 16S rRNA gene (~1,550 bp) was amplified by polymerase chain reaction (PCR) (NZYProof 2X Green Master Mix, NZYTech) and the universal primers 27F and 1492R, incorporated with a 5′ tail sequence (Oxford Nanopore Technologies, UK) for sample barcoding, at 0.4 μM. Purified PCR products were combined in equimolar proportions, and barcoding was performed using the EXP-PBC001 PCR barcoding expansion kit (Nanopore Technologies, Oxford, UK). The DNA library was prepared using the SQK-LSK114 sequencing kit (Nanopore Technologies, Oxford, UK) following the manufacturer’s instructions. A 16S Fastq analysis was performed by uploading the data to the EPI2ME Desktop Agent, version 3.7.3 (Nanopore Technologies, UK).

### 2.4 *In vitro* static digestion of gliadin

**Figure 2:**
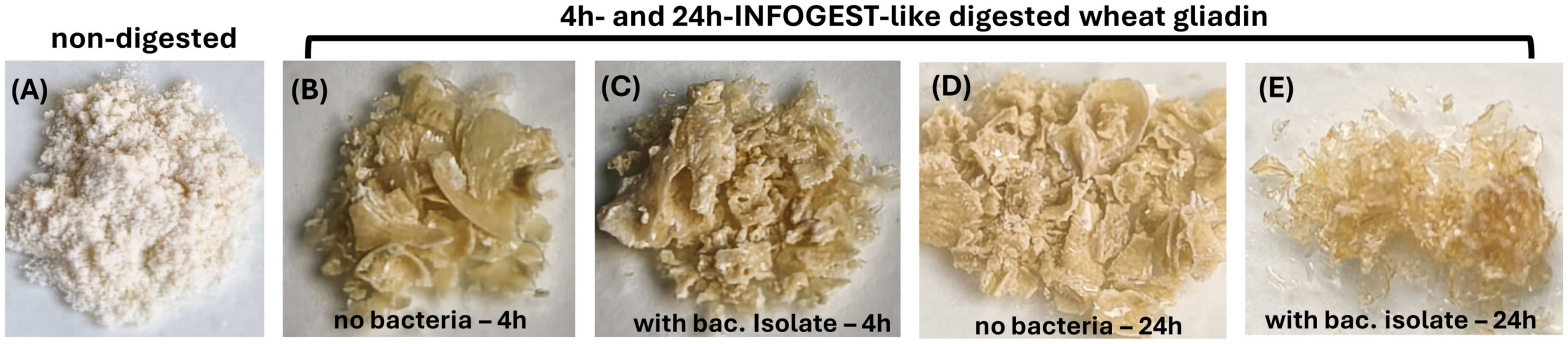
Wheat gliadin powder. **(A)** Non-digested wheat gliadin (Sigma-Aldrich, Germany). Freeze-dried INFOGEST-like digested wheat gliadin (d-gliadin without bacteria) after 4 h **(B)** and 24 h **(D)** of digestion. Freeze-dried INFOGEST-like d-gliadin in the presence of bacterial isolates of *S. marcescens* obtained from stool samples, after 4 h **(C)** and 24 h **(E)** (↓~25% of dry weight after 24 h, relatively to the protein material obtained after 4 h of digestion).

*In vitro* digestions of wheat (*Triticum aestivum*) gliadin (G3375Sigma-Aldrich, Germany—rich in alpha-gliadin, 20–25 kDa, Figure 2A; Supplementary Figure S2) were conducted according to a modified version of the static digestion INFOGEST 2.0 protocol (Brodkorb et al., 2019), focused on the digestion of protein material. A volume (24 mL) of simulated gastric fluid (SGF) (1.25x electrolyte stock solution of 0.5 M KCl, 0.5 M KH_2_PO_4_, 1 M NaHCO_3_, 2 M NaCl, 0.15 M MgCl_2_(H_2_O)_6,_ 0.5 M (NH_4_)_2_CO_3_, and 6 M HCl at pH 3.0) was added to 2 g of gliadin and mixed with 0.3 M of CaCl_2_, 2,000 U/mL of porcine pepsin (P7000, Thermo Fisher, United States), with pH adjustment to 3.0, with 1.0 M HCl. The mixture was incubated at 37 °C, under magnetic stirring at 170 rpm, for 2 h. Then, a volume (24 mL) of simulated intestinal fluid (SIF) (1.25x electrolyte stock solution of 0.5 M KCl, 0.5 M KH_2_PO_4_, 1 M NaHCO_3_, 2 M NaCl, 0.15 M MgCl_2_(H_2_O)_6_, and 6 M HCl at pH 7.0) was added to the gastric chyme to a final ratio of 1:1 (v/v), followed by CaCl_2_, 100 U/mL of porcine trypsin (T0303, Sigma-Aldrich, Germany) and 25 U/mL of bovine chymotrypsin (C4129, Sigma-Aldrich, Germany), at pH 7.0. The mixture was incubated at 37 °C under stirring at 170 rpm for 2 h, followed by an additional 22 h. Control samples were collected after 4 h-(2 h SGF-pepsin + 2 h SIF-chymotrypsin/trypsin) and 24 h-(2 h SGF-pepsin + 22 h SIF-chymotrypsin/trypsin) of digestion. Phenylmethylsulphonyl fluoride (PMSF) was added (at a final concentration of 1 mM) to stop the enzymatic reactions. Samples were centrifuged (10,000 × g, 5 min, 4° C) and supernatants were freeze-dried.

To test the effect of commensal bacteria in gliadin digestion, the bacterial isolates selected (Table 1; Figure 1) were first grown aerobically in tryptic soy broth (TSB) at 37 °C, under agitation (150 rpm) (Thermo Scientific™ MaxQ™ 4450 shaker). Optical density at 600 nm was recorded every half an hour (LLG-uniSPEC 2 spectrophotometer), and cultures were harvested (upon reaching mid-exponential growth) and centrifuged at 5,000 rpm, 10 min. Bacterial pellets of 1 mL of cultures were resuspended in (1 mL + 49 mL) 1.25x SIF with 0.3% (w/v) glucose. Then, cells were grown aerobically at 37 °C, 170 rpm, until mid-exponential growth, and added to gliadin in the intestinal phase (SIF) of the INFOGEST-like digestion -(2 h SGF-pepsin + 2 h SIF-chymotrypsin/trypsin + bacterial isolates). Even if the small intestine is a microaerophilic to anaerobic environment, aerobic conditions coupled with the *in vitro* INFOGEST-like protocol were used for all bacteria. The resulting digesta were collected after 4 h-(2 h SGF-pepsin + 2 h SIF-chymotrypsin/trypsin + bacterial isolates) and 24 h-(2 h SGF-pepsin + 22 h SIF-chymotrypsin/trypsin + bacterial isolates) of incubation, in the presence of the bacterial isolates. PMSF and 0.02% (w/v) sodium azide were added to stop the enzymatic reactions and bacterial growth, respectively. Sodium azide is a potent antimicrobial agent that can interact with proteins, particularly heme proteins (Bellaver et al., 2016; Bouacem et al., 2018). All the 4 h(2 h + 2 h)- and 24 h(2 h + 22 h)-digesta collected were centrifuged (10,000 × g, 5 min, 4° C) to remove cell debris. Supernatants were collected and freeze-dried (Figure 2).

**Table 1:** Sample origin and identification of the selected bacterial isolates.

Sample type	Subject	Bacteria	Origin	Age
Stool	184B	*Pseudomonas aeruginosa*	CeD first-degree relative	80
	228B	*Serratia marcescens*	CeD first-degree relative	64
	254D	*Bacillus tropicus*	CeD first-degree relative	33
	254C	*Bacillus tropicus*	CeD first-degree relative	33
	255B	*Cronobacter sakazakii*	CeD patient	9
	325D	*Enterococcus faecalis*	CeD first-degree relative	74
Blood	CBI317B	*Micrococcus luteus*	CeD patient	39
	CBI304	*Micrococcus luteus*	CeD first-degree relative	55
	CBI144	*Micrococcus* sp.	CeD patient	24

### 2.5 Size-exclusion FPLC

Gliadin digesta (d-gliadin) were analyzed by size-exclusion FPLC using an ÄKTA start™ chromatography system (Cytiva) equipped with a HiLoad 16/600 Superdex 75 prep grade column (Cytiva) (separating range 70 to 3 kDa). The system equilibrated for at least three column volumes (SIF 1.0 mL/min flow rate) before sample injection (2.0 mL at protein concentrations of 0.3 mg/mL). Given that the FPLC chromatograms reported the presence of a mixture of protein populations, the results were grouped according to molecular weight (MW, kDa) in three categories where d-gliadin stands for “digested gliadin”: d-gliadin/peptides large aggregates (>70 kDa), d-gliadin/peptides intermediate aggregates (70–10 kDa), and d-gliadin peptides (<10 kDa).

### 2.6 Fluorescence spectroscopy

Fluorescence spectra were collected using a SpectraMax iD5 microplate reader (Molecular Devices, United States) in black clear-bottom 96-well microplates (NUNC, Denmark), at 25 °C. Intrinsic fluorescence emission spectra were acquired with excitation wavelength at 280 nm and emission from 320 to 420 nm. Thioflavin-T (ThT) emission spectra were recorded with excitation at 440 nm and emission from 480 nm to 600 nm. A ThT stock solution in glycine–NaOH buffer at pH 9.0, was added to the protein solutions (1 mg/mL), to a final ThT concentration of 10 μM.

### 2.7 Congo red binding assay

Samples of the 4 h- and 24 h-digesta of gliadin (5 mg/mL) in the absence and presence of the bacterial isolates were added to a 5 μM Congo red solution (Klunk et al., 1989) prepared in SIF at pH 7.0. UV–Vis spectra (UV500 Spectronic Unicam spectrophotometer, UK) were acquired between 400 and 650 nm. Spectra of buffer solutions in the absence of gliadin were also recorded and used for baseline subtraction.

### 2.8 Dynamic light scattering

Dynamic light scattering (DLS) measurements were performed with a Malvern Zetasizer Nano ZS (Worcestershire, UK), controlled by the Zetasizer software v8.01. Samples were analyzed at 25 °C using 0.3 × 0.3 cm quartz cuvettes. Measurements included four readings at each of the two angles (12.7° and 173°), each consisting of five individual runs of 10 s. A viscosity of 0.95 mPa.s and a refractive index of 1.339, determined experimentally with a Rudolph J157 refractometer (Hackettstown, NJ, United States), were used in data analysis. All buffer solutions were pre-filtered through 0.2 μm filters (Whatman, Maidstone, UK) to avoid contamination by particles external to the experiment. Samples of the 4 h- and 24 h-digesta of gliadin (5 mg/mL) in the absence and presence of the bacterial isolates were analyzed in quadruplicate. Signal areas were integrated to find the percentage of each protein population. Given that the DLS measurements indicated the presence of a mixture of particles in the nanometric and micrometric ranges (>20 nm and <1 μm), the results were grouped by diameter (*d*) into two size categories (*d* of 20–130 nm and *d* > 130 nm).

### 2.9 Fourier transform infrared spectroscopy

Fourier Transform Infrared Spectroscopy (FTIR) experiments were carried out on a ThermoNicolet IR300 spectrometer (Thermo Scientific, Waltham, United States), with a Ge/KBr beam splitter and a deuterated triglycine sulfate (DTGS) detector. Spectra were collected between 3,800 and 400 cm^−1^, with a spectral resolution of 1 cm^−1^, using the KBr pellet technique (Forato et al., 1998).

### 2.10 Scanning electron microscopy

Gliadin aggregates were analyzed by SEM using a Tescan, Vega 3 software. Samples (10 μL) were deposited on uncoated coverslips and allowed to dry and adhere to the surface. Samples were sputter-coated with gold/palladium for 30 and visualized with electron beams at 5 kV and 15 kV.

### 2.11 Enzyme-linked immunosorbent assays

The antigenicity of the gliadin digesta was evaluated by ELISAs against anti-gliadin (wheat)–polyclonal antibodies, as well as against R5 and G12 monoclonal antibodies.

#### 2.11.1 Polyclonal anti-gliadin

Protein samples (5 mg/mL) were added to 50 mM carbonate buffer, pH 9.4, and coated overnight, at 4 °C. Wells were blocked with 5.0% (w/v) bovine serum albumin for 1 h, at room temperature. Anti-gliadin (wheat)–peroxidase conjugate polyclonal antibodies produced in rabbit (Sigma-Aldrich, 1:1000 dilution) were added to the wells and left to react for 2 h, at room temperature. Wells were washed between steps with phosphate buffer, 0.05% (v/v) Tween-20, pH 7.4. Color development was achieved with 1 mM 2,2′-azino-di-(3-ethylbenzthiazoline sulfonic acid), in 20 mM phosphate/citrate buffer, pH 4.1, and 0.02% (v/v) H_2_O_2_, at room temperature. Optical density was measured after 15 min at 405 nm using a SpectraMax iD5 microplate reader (Molecular Devices, United States). Experiments were performed three times with samples analyzed in quadruplicate.

#### 2.11.2 Monoclonal R5 and G12

RIDASCREEN Gliadin R5 kits [R7001, R-Biopharm, Germany—monoclonal IgG2b antibodies against the QQPFP sequence found in gliadin (Osman et al., 2001; Kahlenberg et al., 2006)] and AgraQuant gluten G12 kits [Romer Labs, Austria—monoclonal antibodies against the QPQLPY sequence (Morón et al., 2008)] were used to perform sandwich ELISAs according to the manufacturers’ instructions. Absorbances were measured at 450 nm, within 10 min, and expressed as ng/mL (R5) and μg/mL (G12). Experiments were performed three times for each detection kit with samples analyzed in quadruplicate.

### 2.12 Statistical analysis

Experiments were performed at least in triplicate. Quantitative variables were reported via means and standard deviations. Data were analyzed by analysis of variance (ANOVA) to determine differences between means, deemed statistically significant at *p* < 0.05. One-way ANOVAs were used to perform comparisons among the 4 h- and 24 h-experimental groups, while two-way ANOVAs were used to perform comparisons between time points (4 h and 24 h) and digestions in the absence and presence of bacteria. *Post-hoc* test outcomes were derived from Tukey’s test for cases of homogeneous variance (with *p < 0.05, **p < 0.01, and ***p < 0.001). The Benjamini–Hochberg procedure was also used to correct for multiple comparisons.

## 3 Results and discussion

### 3.1 Gliadin-digestion by commensal bacteria from CeD patients

A total of 369 stool and 23 blood bacterial isolates were obtained from samples collected from CeD patients, under a gluten-free diet, and from their non-CeD first-degree relatives (Roque et al., 2024). Among those, 79 randomly selected stool isolates and all 23 blood isolates were tested for their *in vitro* capacity to degrade gliadin. Out of the 102 bacterial isolates tested, 12 stool and 6 blood isolates showed gliadin-degrading activity, among which 6 from stool and 3 from blood presented a halo ≥ 5 mm around bacterial colonies (Figure 1). These 9 isolates were selected for subsequent analyses: the Gram-positive *Bacillus tropicus, Enterococcus faecalis, Micrococcus* sp., and *Micrococcus luteus,* and the Gram-negative *Cronobacter sakazakii, Serratia marcescens,* and *Pseudomonas aeruginosa*. Three isolates were obtained from CeD patients (stool—*Cronobacter sakazakii* and blood—*Micrococcus* sp. and *Micrococcus luteus* CBI317B), and the remaining from non-CeD first-degree relatives. Although of different origin, some isolates were of the same species (*Bacillus tropicus* 254C and 254D; *Micrococcus luteus* CBI317B and CBI304).

So far, *B. tropicus*, *M. luteus*, *C. sakazakii,* and *S. marcescens* have not been directly related to gluten intolerance and/or celiac disease patients. *Bacillus tropicus* is a Gram-positive, spore-forming, facultative anaerobic bacterium that, due to its chromium-reducing ability, holds potential to be used for bioconversion and bioremediation (Tuli et al., 2024). *M. luteus* is also a Gram-positive bacterium that has already been used as a probiotic for fish (Abd El-Rhman et al., 2009). *M. luteus* bloodstream infections are of low incidence and are mainly associated with immunocompromised patients or patients who underwent surgery (Zhu et al., 2021). In turn, *C. sakazakii* is a Gram-negative global food-borne opportunistic pathogen that has been associated with fatal systemic infections in neonates (via contaminated powdered formula), infants, elders, and immune-compromised patients (Phair et al., 2022). *S. marcescens* is also a Gram-negative bacterium frequently found in water, soil, and animal intestines, with potential to be used in sustainable pest management (Akhtar et al., 2025). In humans, *S. marcescens* has been associated with the development of peritonitis (Yang and Li, 2020). Hence, since *C. sakazakii* and *S. marcescens* are opportunistic pathogens that can seriously damage the intestinal barrier and invade epithelial tissues, these bacteria cannot be used as probiotics.

On the other hand, with respect to *P. aeruginosa* and *E. faecalis*, some studies have already reported their involvement in microbe–gluten–host interactions. Small intestinal bacteria have been shown to work as gluten/gliadin degraders, via different proteolytic mechanisms, which can increase or decrease peptide immunogenicity in genetically predisposed individuals. *P. aeruginosa* is an opportunistic pathogen, already identified as a gluten-degrader, due to elastase (Rahmani et al., 2024) and pseudolysin activities (Caminero et al., 2016; Wei et al., 2015). Hence, *P. aeruginosa* may work in conjunction with gluten to trigger and escalate intestinal inflammation (Rossi et al., 2023; Caminero et al., 2019). Similarly, *E. faecalis* has also previously shown gluten-degrading activity (Capuani et al., 2013; El Mecherfi et al., 2022) associated with the expression of certain proteases (M'hir et al., 2009) such as zinc metalloproteases (Le Corre et al., 2025). Hence, this group of gliadin degraders (Table 1; Figure 1) was selected to be further tested in the wheat gliadin *in vitro* INFOGEST-like digestions carried out.

### 3.2 *In vitro* INFOGEST-like digestion of wheat gliadin and FPLC analysis

Wheat gliadin digestions were performed according to a static INFOGEST-like protocol (see Materials and Methods). First, digestions were carried out with wheat gliadin, solely, in the absence of bacteria (control digestions). After digestion, the gliadin digested (d-gliadin) samples still presented a large amount of undigested material (Figure 2B) and were analyzed by FPLC to detect peptides within the range of 3 to 70 kDa. As expected, gliadin was not considerably cleaved by pepsin (Supplementary Figure S3A) (Herrera et al., 2021), but was partially digested by chymotrypsin and trypsin, forming smaller peptide fragments (Herrera and Dodero, 2021) (Supplementary Figure S3B). Supplementary Figure S3D shows the overlay of the FPLC chromatograms of the 4 h- and 24 h-digesta of d-gliadin in the absence of bacteria. Moreover, considering that in most healthy human organisms, feces remain in the intestine/colon for approximately 24 h (16–30 h for colonic transit times, 23–37 h for whole gut transit time) (Nandhra et al., 2023; Procházková et al., 2023), the proteolytic state of gliadin was also analyzed after 24 h of incubation with the bacterial isolates. Since bacteria can activate the expression and secretion of additional proteases into the extracellular space, during longer incubation periods, a 24-h sampling was also carried out to investigate the proteolytic state of d-gliadin.

The d-gliadin peptides (<10 kDa) and d-gliadin/peptides large aggregates (>70 kDa) detected after 4 h of digestion, augmented considerably after 24 h, indicating that protein digestion continued and that the aggregates/oligomers detected (40 min, MW > 70 kDa) could have either resulted from cleavage/dissociation of larger protein clusters of undigested material (Urade et al., 2018), and/or from aggregation of the d-gliadin peptides/fragments formed (Freitas et al., 2022), as previously reported for aggregates of the 33-mer gliadin peptide (Herrera et al., 2014).

### 3.3 Chemical nature of INFOGEST-like digested wheat gliadin

SEM, DLS, FTIR, and fluorescence spectroscopy were used to characterize the d-gliadin digesta. All fractions exhibited typical protein/tryptophan fluorescence emission spectra (Supplementary Figure S4A) but showed different maximum emission wavelengths (λ_max_). Fraction A of the 4 h d-gliadin, and fractions A and B (λ_max_ 370 nm) of the 24 h d-gliadin [mainly composed of d-gliadin intermediate aggregates (70–10 kDa); and d-gliadin peptides (<10 kDa)] presented maximal emission wavelengths blue-shifted toward lower wavelengths (from 365 nm to 355 nm), reporting chemical environments of tryptophan residues less exposed to the solvent, as expected for protein aggregates (Vaz et al., 2024).

**Figure 3:**
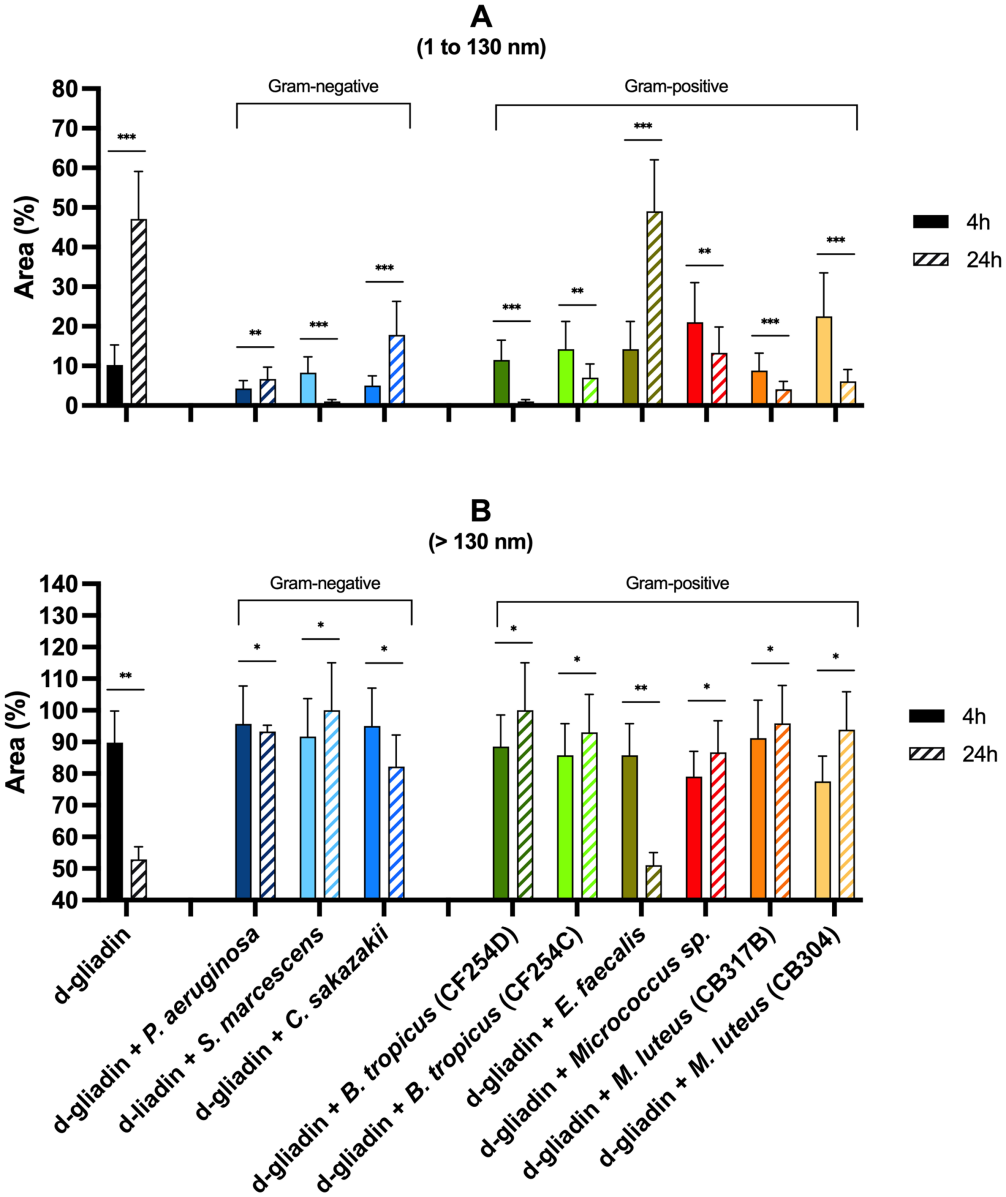
DLS profiles of 4 h- and 24 h-INFOGEST-like d-gliadin (digested gliadin) in the absence and presence of the bacterial isolates: *P. aeruginosa*, *S. marcescens*, *C. sakazakii*, *B. tropicus*, *E. faecalis*, *Micrococcus* sp., and *M. luteus.* DLS data are reported in two size categories according to diameter (*d*) (**A**: 1–130 nm and **B**: >130 nm). ANOVA and Tukey’s *post hoc* tests were used to identify significant differences among groups (*p < 0.05, **p < 0.01, and ***p < 0.001).

In addition, to detect larger protein clusters that would not be detected by FPLC analysis, the 4 h- and 24 h-digesta were also analyzed by DLS (Figure 3; Supplementary Figure S4). After 4 h of digestion, the DLS profile detected a major population of large particles/clusters (~600 nm in diameter, *d*), as previously seen by other authors for d-gliadin (Herrera et al., 2018; Herrera et al., 2021), along with two other minor populations of smaller size (*d*, ~40 and ~100 nm) (Supplementary Figure S4 – 4 h d-gliadin). In opposition, after 24 h of digestion, the DLS profile was no longer dominated by larger protein particles/clusters and showed a more heterogeneous profile with smaller entities (*d*, 20–400 nm) (Supplementary Figure S4 – 24 h d-gliadin). These results corroborate the FPLC data for the 24 h-digesta that also showed an increase in smaller d-gliadin peptides (~5 kDa) after 24 h. These differences were also confirmed by SEM, where, after 4 h of digestion, a pattern of spherical shapes (1–10 μm) of the poorly digested d-gliadin, like the spherical forms of “microcellular foam” formed by dried gliadin powder (Quester et al., 2014), was detected. Conversely, after 24 h of incubation, smaller, square-shaped structures could be observed (Supplementary Figure S5B). These square-like/rhombic structures could have resulted from the presence of more d-gliadin peptides (e.g., 33-mers, 25-mers, and/or 13-mers) that self-assembled/oligomerized into larger particles, as previously seen for gliadin protofilaments (Herrera et al., 2018; Herrera et al., 2018; Herrera et al., 2014; Herrera et al., 2015; Herrera et al., 2016; Herrera et al., 2020), in a process dependent on concentration, ionic strength, and pH, given their behavior as hydrophobic colloids with amyloid-like character, as also tested here by the ThT fluorescence (Supplementary Figure S6B), Congo Red binding (Supplementary Figure S6C) and FTIR analyses (Supplementary Figure S6A) carried out here.

ThT is known to work as a protein structure indicator given its ability to increase its fluorescence intensity when bound to stacked elements of β-sheet amyloid-like structures (Almeida and Brito, 2020; Lambrecht et al., 2021; Feng et al., 2024). Thus, to analyze the structural nature of the d-gliadin oligomers/aggregates present in the 4 h- and 24 h- digesta, a ThT binding assay was performed. In the absence of gliadin, or in the presence of non-digested gliadin, ThT presented no fluorescence, with a spectrum of very low intensity, typical of the free dye in solution (Supplementary Figure S6B), whereas in the presence of the 4 h- and 24 h-gliadin digesta, the bound ThT-dye fluoresced greatly, especially with the 24 h-digesta (Supplementary Figure S6B). This ThT-binding ability exhibited by gliadin agrees with previously reported data, which stated that trypsin-hydrolyzed gluten, glutenin, and especially gliadin peptides were able to gradually self-assemble into amyloid-like fibrils after 36 h of incubation (Feng et al., 2024). Similarly, the Congo Red binding assay showed positive binding. Congo Red is another specific dye widely used to detect cross-beta sheet structures in amyloid aggregates (Klunk et al., 1989). The binding of Congo Red to amyloid structures induces optical changes associated with a red shift (~15 nm) of the maximum absorbance wavelength (λ_max_), accompanied by the appearance of a second shoulder/peak (at ~540 nm) (Espargaró et al., 2020), as seen in Supplementary Figure S6C. The absorption spectrum of the free dye presented a λ_max_ at 485 nm, which, in the presence of d-gliadin samples (especially the 24 h-digesta), shifted toward higher wavelengths (500 nm), also exhibiting a second shoulder peak at ~540 nm, as previously seen for protein aggregates of white bread (Yadav et al., 2023) and wheat gluten, glutenin and gliadin (Liang et al., 2024). Moreover, the FTIR analyses also confirmed an increase in β-sheet structure (after 24 h), particularly associated with the Amide I band between 1,600–1,700 cm^−1^ (assigned to C=O bond stretching coupled with C-N stretching and N-H bending), also present in the amyloid structures formed by the Abeta-peptides (amyloid β_1-42_ and amyloid β_1-42_), alpha-synuclein, and prion protein PrP_82-146_ (Wilkosz et al., 2020).

Hence, in sum, after 4 h of digestion, d-gliadin is still poorly digested (Figure 2) and presents intermediate and large protein clusters in solution; while after 24 h of incubation, d-gliadin peptide fragments dominate and exhibit a tendency to aggregate into fibril structures (Supplementary Figures S4–S6).

### 3.4 Effect of the gliadin-degrading commensal bacteria

**Figure 4:**
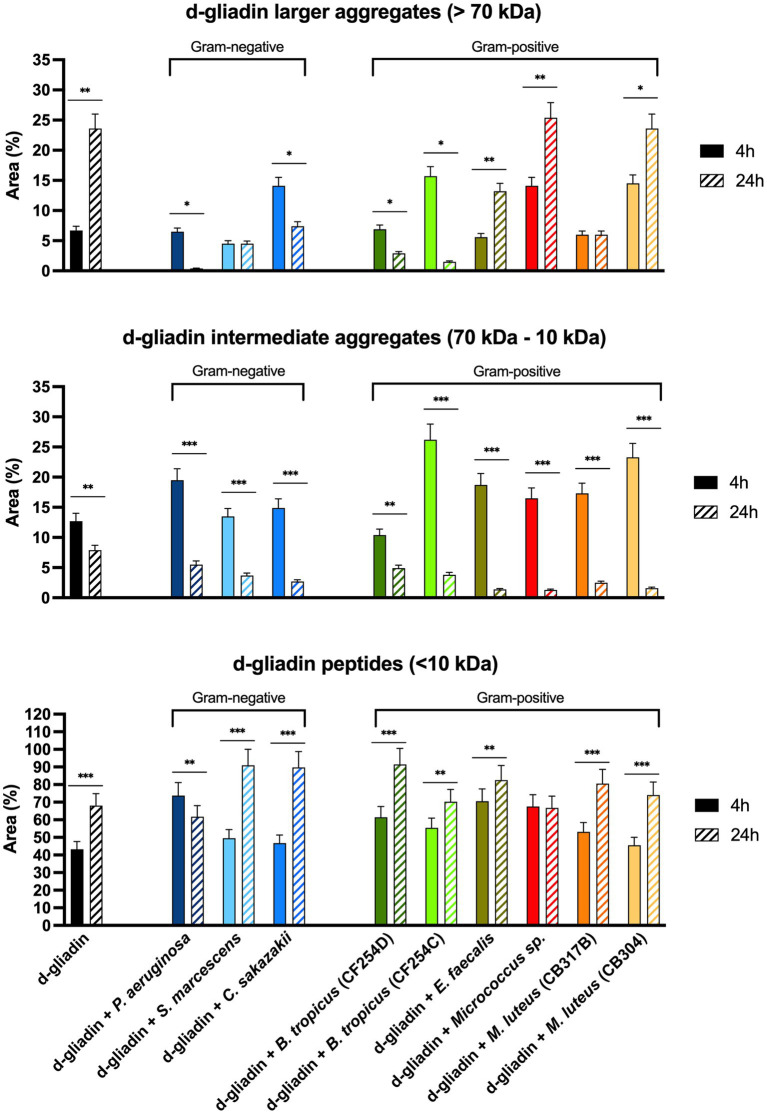
Protein populations of the FPLC chromatograms of 4 h- and 24 h-INFOGEST-like d-gliadin (digested gliadin) in the absence and presence of the bacterial isolates: *P. aeruginosa*, *S. marcescens*, *C. sakazakii*, *B. tropicus*, *E. faecalis*, *Micrococcus* sp., and *M. luteus*. FPLC data are reported in three molecular weight categories (>70 kDa – d-gliadin larger aggregates; 70 kDa–10 kDa – d-gliadin intermediate aggregates/oligomers; and <10 kDa – d-gliadin peptides). ANOVA and Tukey’s *post hoc* tests were used to identify significant differences among groups (*p < 0.05, **p < 0.01, and ***p < 0.001).

**Figure 5:**
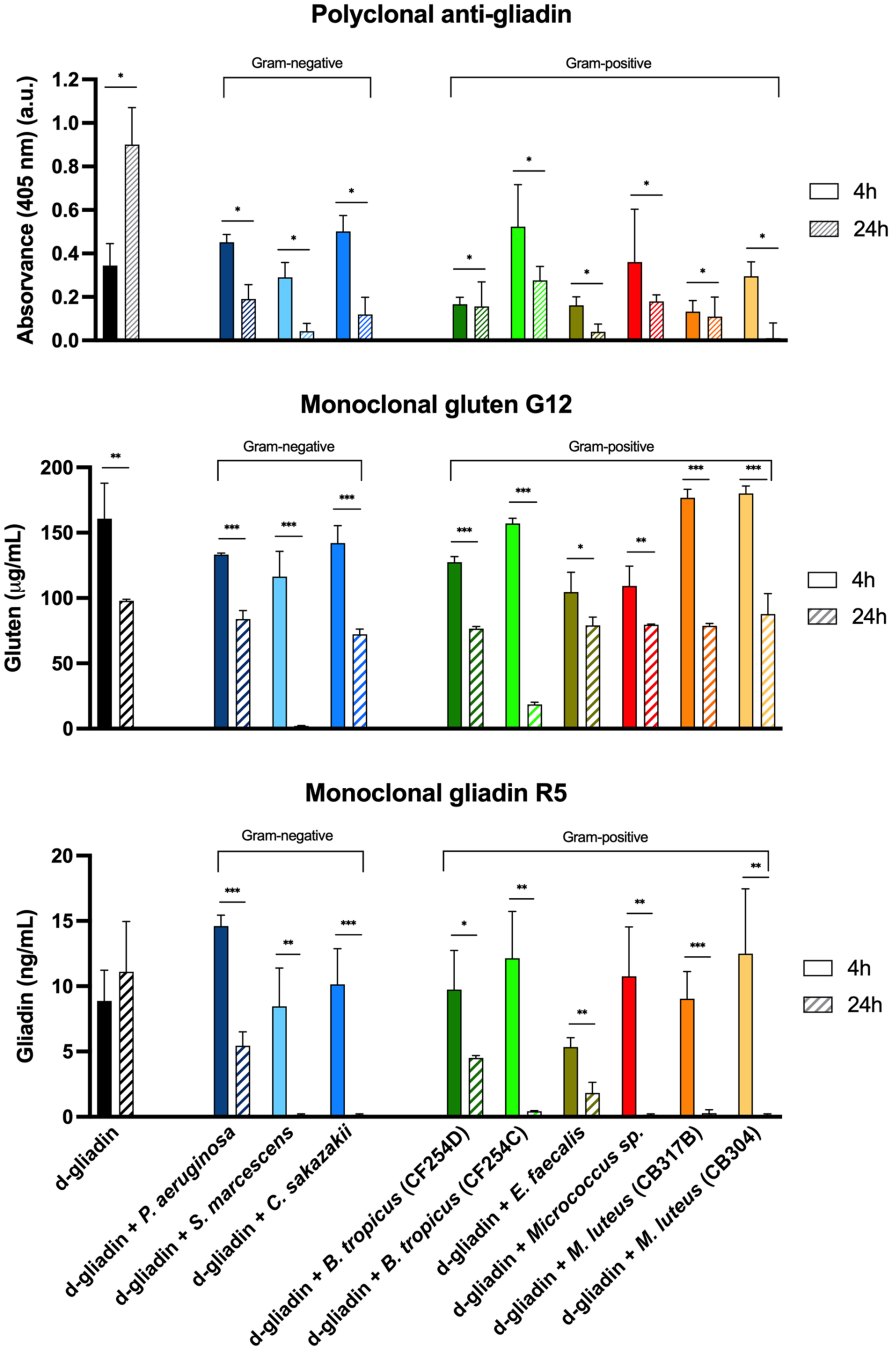
ELISA assays against polyclonal anti-gliadin and monoclonal R5 and G12 monoclonal antibodies of the 4 h- and 24 h-INFOGEST-like d-gliadin (digested gliadin) in the absence and presence of the bacterial isolates: *P. aeruginosa*, *S. marcescens*, *C. sakazakii*, *B. tropicus*, *E. faecalis*, *Micrococcus* sp., and *M. luteus.* ANOVA and Tukey’s *post hoc* tests were used to identify significant differences among groups (*p < 0.05, **p < 0.01, and ***p < 0.001).

Following proteolytic digestion, bacterial isolates were added. After 4 h of digestion in the presence of the bacterial isolates, although still presenting d-gliadin large (>70 kDa) and intermediate aggregates (70–10 kDa), all FPLC chromatograms (Figure 4; Supplementary Figure S7) detected an increase in d-gliadin peptides (<10 kDa), attesting for the additional cleavage performed by the bacteria, particularly by the bacterial isolates of *P. aeruginosa, B. tropicus, E. faecalis, Micrococcus* sp., and *M. luteus* CBI304, when compared to control—d-gliadin (without bacteria). This proteolytic activity exhibited by some bacteria had already been reported for *P. aeruginosa* (Caminero et al., 2016; Wei et al., 2015) and *E. faecalis* (El Mecherfi et al., 2022), but not for *B. tropicus*, *C. sakazakii*, *S. marcescens*, or *Micrococcus luteus*. Moreover, depending on the bacteria, different amounts of fragments and oligomers/aggregates were observed, suggesting that different metabolisms and/or digestion kinetics took place, especially with *P. aeruginosa*, *C. sakazakii*, *M. luteus* CBI304, and *B. tropicus* 254C, which led to higher amounts of d-gliadin peptides and aggregates already after 4 h of digestion. In turn, after 24 h, when compared to digestions without bacteria, most chromatograms (Figure 5; Supplementary Figure S8) showed higher amounts of d-gliadin peptides and fewer aggregates, particularly in the case of *P. aeruginosa*, *B. tropicus*, *C. sakazakii*, *M. luteus*, and *S. marcescens*. In addition, the 24 h d-gliadin produced in the presence of *Micrococcus* bacteria (*Micrococcus* sp. and *Micrococcus luteus*) presented larger amounts of d-gliadin aggregates (>70 kDa), when compared to 24 h-digesta by other bacteria or with no bacteria (Figure 5; Supplementary Figure S9).

Similarly, the DLS profiles of the 4 h- and 24 h-digesta obtained in the presence of bacteria (Figure 3; Supplementary Figure S9) also pointed out differences between the digestions with and without bacteria (control), as well as amongst different bacteria. In the presence of gliadin-degrading microbes, the larger d-gliadin aggregates detected in the 4 h-digesta (*d*, ~600 nm) were no longer present, and the samples were mostly populated by smaller entities (*d* < 300 nm) in all cases, confirming the additional degrading effect of the bacteria. However, after 24 h, a different pattern was observed. Incubations in the presence of *C. sakazakii*, *Micrococcus*, *M. luteus* CBI304 and *B. tropicus* 254C, beyond presenting smaller particles (*d* ~ 50 nm), also led to the formation of larger entities (*d* ~ 400 nm) after 24 h, as confirmed by the presence of the remaining insoluble material (Figure 2, Supplementary Figure S5, ↓ ~ 25% of protein material relatively to the protein material obtained after 4 h of digestion) that could have resulted from the self-assembling/aggregation of the d-gliadin bacteria-undigested fragments with time.

Hence, to structurally characterize the oligomers/aggregates formed upon digestion in the presence of the bacterial isolates, ThT fluorescence and Congo Red binding assays were also carried out. All intrinsic fluorescence spectra presented typical profiles of protein emission, with λ_max_ at 360 nm. Additionally, all 4 h d-gliadin bacterial digesta exhibited positive ThT fluorescence emission (Supplementary Figure S10A) and red-shifted Congo Red absorbance spectra (Supplementary Figure S10B). Moreover, after 4 h of digestion, the bacterial isolates that led to the production of more d-gliadin aggregates (than without bacteria) as seen by DLS and FPLC (Figures 3, 4), were also the same that led to more positive signals of ThT fluorescence and Congo Red binding (Supplementary Figure S10), namely *C. sakazakii*, *M. luteus* CBI304, *B. tropicus* 254C and especially the *Micrococcus* sp., as also confirmed by SEM (Supplementary Figure S5) where rod-like structures (*d* ~ 100 nm) formed long branched assemblies (length > 20 μm), as previously seen for gliadin fragments adsorbing onto mica surfaces (Herrera et al., 2018; Herrera et al., 2014; Herrera et al., 2015; Herrera et al., 2016; Herrera et al., 2020).

However, after 24 h of incubation in the presence of the various bacterial isolates, a decrease in ThT was observed in all cases, indicating that the insoluble undigested d-gliadin material remaining in solution was able to aggregate into less ordered/amorphous spherical structures (SEM – Supplementary Figure S5) than control digestions without bacteria, especially in the case of *P. aeruginosa* (with the lowest ThT signals and Congo Red redshifts), also presenting a low fraction of d-gliadin aggregates in the FPLC chromatograms after 24 h. These results indicate that the 4 h-digesta were further cleaved by the bacterial isolates into remaining undigested d-gliadin peptides (~5 kDa), that aggregated along time (24 h) to form more amorphous material (*d* > 130 nm, Figure 3), showing less ThT and Congo Red positivity (Supplementary Figure S10) and β-sheet enrichment (as also seen by the general reduction of the Amide I absorbance from a range of 0.20–0.63 to a range 0.10–0.58) (Supplementary Figure S11). This ability of wheat gliadin to form different nanostructures in solution, depending on fragment length, concentration, ionic strength, and pH, has already been detected by other authors who observed micelle-type nanostructures with higher zeta potential, at pH 3.0; while at pH 7.0, amorphous microaggregates with lower zeta potential were found (Herrera et al., 2016). These microaggregates found at pH 7.0 were also unable to bind the highly hydrophobic fluorescent probe Nile Red (Herrera et al., 2016), likely due to the occlusion of hydrophobic regions in the protein–protein interfaces formed during aggregation. Interestingly, this aggregation ability of gliadin has also been used to form electro-spun nanofibers to be used as non-functional packaging systems loaded with bioactive ingredients (e.g., essential oils) (Bahrami et al., 2022).

Moreover, because different fragments and aggregates/nanostructures can interact differently with the intestinal mucosa and potentially trigger and/or exacerbate inflammatory responses in sensitive individuals, the antigenic profiles of the digesta were analyzed by ELISA.

### 3.5 Antigenic nature of the protein digesta

The antigenicity of the gliadin digesta was evaluated by three ELISA assays, namely, against anti-gliadin polyclonal antibodies, as well as against monoclonal antibodies (R5 and G12) (Figure 5). Control digestions (d-gliadin with no bacteria) yielded positive results by the polyclonal ELISA, indicating that the d-gliadin peptides (<10 kDa) and/or the subsequent larger oligomers/aggregates (>70 kDa) formed over time, presented high antigenicity. Conversely, in the presence of bacterial isolates, the antigenicity presented by d-gliadin diminished with time (Figure 5). When compared to control (without bacteria), some bacterial isolates led to higher antigenicity after 4 h of digestion (i.e., *P. aeruginosa*, *C. sakazakii*, and *B. tropicus* 254C), while the remaining led to a lower antigenic response (i.e., *B. tropicus* 254D, *E. faecalis*, and *S. marcescens*). This higher antigenic behavior induced by the presence of bacterial isolates of *P. aeruginosa* agrees with previous data that showed that d-gliadin peptides degraded by *P aeruginosa*, during 4 h, at 37 °C, activated the immune system of germ-free C57BL/6 mice, by producing peptides that better translocated the mouse intestinal barrier (Caminero et al., 2016; Caminero et al., 2019). Conversely, after 24 h of incubation with the bacterial isolates, a different pattern was observed (Figure 5). All digesta led to lower antigenicity than at 4 h, as well as to lower antigenicity than control (d-gliadin), particularly in the case of the bacterial isolates of *E. faecalis, S. marcescens*, and *M. luteus* CBI304. These results also correlate with the SEM, FTIR, ThT, and Congo Red data, which showed lower ThT intensities, less redshifted Congo Red spectra (Supplementary Figure S10), and less β-sheet enrichment (Supplementary Figure S11), after 24 h of incubation with bacteria. In sum, after 4 h of digestion, the d-gliadin peptides and/or as ThT/Congo Red-positive aggregates exposed more antigenic epitopes; while the insoluble d-gliadin amorphous structures formed after 24 h (Figure 2; Supplementary Figure S5) led to lower ThT/Congo Red-positivity and β-sheet enrichment with lower antigenic potential by the polyclonal anti-gliadin ELISA assay.

Moreover, the results obtained with the polyclonal anti-gliadin antibodies were supported by the monoclonal R5 and G12 ELISAs. After 4 h of gliadin digestion in the presence of the bacterial isolates, the antigenic response detected by the R5 and G12 antibodies was similar to or higher than that for control digestions (d-gliadin without bacteria). In the case of the G12-ELISA, *B. tropicus* CF254C and *M. luteus* also led to the production of d-gliadin peptides with higher antigenic responses, likely due to the exposure of the QPQLPY epitope. Similarly, in the R5-ELISA, the proteolytic activity of *B. tropicus* CF254C, *Micrococcus* sp., and *P. aeruginosa* on d-gliadin also led to higher antigenic responses.

Conversely, different profiles were observed after 24 h. In the absence of bacteria, the antigenic behavior of the d-gliadin peptides and/or aggregates increased in the R5-ELISA (as seen with the polyclonal ELISA), pointing toward the exposure of the QQPFP epitope (present in the 25-mer). In turn, in the presence of the bacterial isolates, the anti-R5 antigenic behavior of d-gliadin reduced considerably in all cases (as in the polyclonal ELISA), suggesting the occurrence of the cleavage/absence of the 25-mer or involvement of this fragment in the formation of insoluble amorphous aggregates, not exposing the QQPFP antigenic sequence. Conversely, the G12-antigenic behavior of the d-gliadin peptides and/or aggregates was higher after 4 h of digestion, but diminished after 24 h of incubation, in both the absence and presence of the bacterial isolates. This decrease in antigenicity after 24 h suggests lower concentrations of the 33-mer in solution, or the involvement of this fragment in the formation of insoluble amorphous material, not exposing the QPQLPY antigenic sequence.

Interestingly, the bacterial isolates obtained from celiac patients (blood – Micrococcus and stool – *C. sakazakii*) showed the smallest digestion halos (*Micrococcus* sp. – 7 mm; *M. luteus* – 5 mm; *C. sakazakii* – 9 mm, Figure 1), as well as led to the formation of large aggregates (>70 kDa) in amounts similar to 4 h-control digestions (without bacteria). Furthermore, the 4 h digesta incubated with these bacterial isolates (*Micrococcus* sp., *M. luteus*, and *C. sakazakii*) also showed positive ThT fluorescence spectra, presence of beta-sheet enrichment (as judged by the FTIR data), and similar or higher antigenicity by the monoclonal antibody G12, relative to control (d-gliadin), suggesting that these aggregates are exposing the QPQLPY epitope (present in the 33-mer), even if after 24 h of incubation the antigenicity was significantly reduced (*p* < 0.001) (Figure 5).

## 4 Conclusion

*In vitro* static digestions of wheat gliadin resulted in the formation of an antigenic protein mixture (peptides and aggregates). Yet, in the presence of gliadin-degrading bacterial isolates, additional proteolytic cleavage occurred, increasing the levels of d-gliadin peptides. After 4 h of digestion, some bacterial isolates led to higher antigenicity than control (d-gliadin without bacteria), due to exposure of the QQPFP and QPQLPY antigenic sequences (i.e., *P. aeruginosa*, *C. sakazakii*, and *B. tropicus* CFI254C), while others led to lower antigenic responses (i.e., *E. faecalis*). Even so, independently of the digestion, metabolism, and/or digestion kinetics executed by each bacterial isolate, all gliadin fragments produced were able to assemble and form ThT/Congo Red-positive and β-sheet-enriched aggregates, after 4 h of digestion. Conversely, after 24 h of incubation in the presence of the bacterial isolates, more fragments were formed, which were also able to aggregate into insoluble amorphous material with less antigenicity (due to lower amounts of the 25- and 33-mers in solution, or involvement of these peptides in forming amorphous aggregates, not exposing antigenic sequences). Therefore, further molecular analyses will be needed to: (I) identify the different fragments formed and the specific gliadin-proteolytic enzymes expressed, (by the bacterial metabolism and/or secreted to the extracellular space, under aerobic and anaerobic conditions); as well as to (II) determine the inflammatory and immunogenic effect caused by these d-gliadin peptides and aggregates to model cells (e.g., Caco-2 cells to simulate the human intestinal epithelium and T-cell assays to test for immunogenicity).

## Data Availability

The datasets presented in this study can be found in online repositories. The names of the repository/repositories and accession number(s) can be found in the article/Supplementary material.
