# Correction: Influence of commensal bacteria on the proteolytic and antigenic profiles of INFOGEST-like digested wheat gliadin

**DOI:** 10.3389/fmicb.2026.1930765

**Published:** 2026-07-21

**Authors:** Flávio Pereira-Costa, Vanessa S. Domingues, Ana Roque, Zaida L. Almeida, Pedro F. Cruz, Rachel Cordeiro, Daniela Trindade, Carla Moura, Joana B. Melo, Sónia G. Pereira, Daniela C. Vaz

**Affiliations:** 1 CiTechCare, Centre for Innovative Care and Health Technology, School of Health Sciences, Polytechnic Institute of Leiria, Leiria, Portugal; 2 Department of Life Sciences, NOVA School of Science and Technology, Universidade NOVA de Lisboa, Caparica, Portugal; 3 Department of Chemistry, Coimbra Chemistry Centre (CQC), Institute of Molecular Sciences (IMS), University of Coimbra, Coimbra, Portugal; 4 School of Health Sciences, Polytechnic Institute of Leiria, Leiria, Portugal; 5 CDRsp, Centre for Rapid and Sustainable Product Development, Polytechnic of Leiria, Marinha Grande, Portugal; 6 Veterinary Clinics Department, Abel Salazar Biomedical Sciences Institute (ICBAS), University of Porto, Porto, Portugal; 7 Polytechnic University of Coimbra, Coimbra, Portugal; 8 Research Centre for Natural Resources, Environment and Society (CERNAS), Polytechnic University of Coimbra, Coimbra, Portugal; 9 Cytogenetics and Genomics Laboratory and iCBR-CIMAGO, Faculty of Medicine, University of Coimbra, Coimbra, Portugal; 10 Associate Laboratory LSRE-LCM, Laboratory of Separation and Reaction Engineering and Laboratory of Catalysis and Materials, Polytechnic Institute of Leiria, Leiria, Portugal

**Keywords:** alpha-gliadin, antigenic gliadin fragments, celiac disease, gliadin-degrading bacteria, INFOGEST *in vitro* digestion, protein aggregates, proteolytic cleavage, anti-gliadin ELISA

In the **Funding** statement, the funder “2022 Beyond Celiac Established Investigator Award (beyondceliac.org)” was omitted. The correct **Funding** statement reads:

“The author(s) declared that financial support was received for this work and/or its publication. This research was supported by a 2022 Beyond Celiac Established Investigator Award (beyondceliac.org). Authors also acknowledge funding from the Coimbra Chemistry Centre—Institute of Molecular Sciences (CQC IMS) which is supported by the Fundação para a Ciência e a Tecnologia (FCT), Portuguese Agency for Scientific Research. CQC is funded by FCT through projects https://doi.org/10.54499/UID/PRR/00313/2025 and https://doi.org/10.54499/UID/00313/2025 and IMS through special complementary funds provided by FCT (https://doi.org/10.54499/LA/P/0056/2020). The financial support of Fundação para a Ciência e Tecnologia (FCT/MCTES) to Vanessa Santos Domingues (UI/BD/150925/2021), Ana Roque (10.54499/UI/BD/151038/2021), Sónia Gonçalves Pereira (10.54499/CEECINST/00051/2018/CP1566/CT0004), ciTechCare (10.54499/UIDB/05704/2020), LSRE LCM (10.54499/UIDB/50020/2020 and 10.54499/UIDP/50020/2020), ALiCE (10.54499/LA/ /0045/2020) and CDRsp (10.54499/UIDB/04044/2020 & 10.54499/UIDP/04044/2020) is also acknowledged.”

The original version of this article has been updated.

