# Supplementary material for: Influence of commensal bacteria on the proteolytic and antigenic profiles of INFOGEST-like digested wheat gliadin
Source: Front Microbiol. 2026 Jul 6;17:1842801. doi: 10.3389/fmicb.2026.1842801 (PMC13384009; doi:10.3389/fmicb.2026.1842801)
Supplement: Supplementary file 1 [file Supplementary_file_1.pdf]

SUPPLEMENTARY MATERIAL

Influence of commensal bacteria on the proteolytic and antigenic profiles of INFOGEST-like digested wheat gliadin

Figure S1. Microbiological screening of bacterial isolates with gliadin-degrading activity.

Figure S2. Coomassie Brilliant Blue stained SDS-PAGE gel of wheat (*Triticum aestivum*) gluten and gliadin.

Figure S3. Size-exclusion FPLC chromatograms of controls and of INFOGEST-like 4 h- and 24 h-digesta of wheat gliadin.

Figure S4. INFOGEST-like 4 h- and 24 h-digesta of wheat gliadin: Intrinsic fluorescence spectra and DLS profiles.

Figure S5. SEM images of 4 h- and 24 h-d-gliadin in the absence and presence of bacteria.

Figure S6. INFOGEST-like 4 h- and 24 h-digesta of wheat gliadin: FTIR, ThT fluorescence and Congo red absorbance spectra.

Figure S7. Size-exclusion FPLC chromatograms of 4 h-d-gliadin in the absence and presence of bacteria.

Figure S8. Size-exclusion FPLC chromatograms of 24 h-d-gliadin in the absence and presence of bacteria.

Figure S9. DLS profiles of 4 h- and 24 h-d-gliadin in the absence and presence of the bacterial isolates.

Figure S10. ThT fluorescence and Congo red spectra of 4 h- and 24 h-d-gliadin in the absence and presence of the bacterial isolates.

Figure S11. FTIR spectra of 4 h- and 24 h- d-gliadin in the absence and presence of the bacterial isolates.

A. No degrading activity **B.** Gliadin-degrading activity

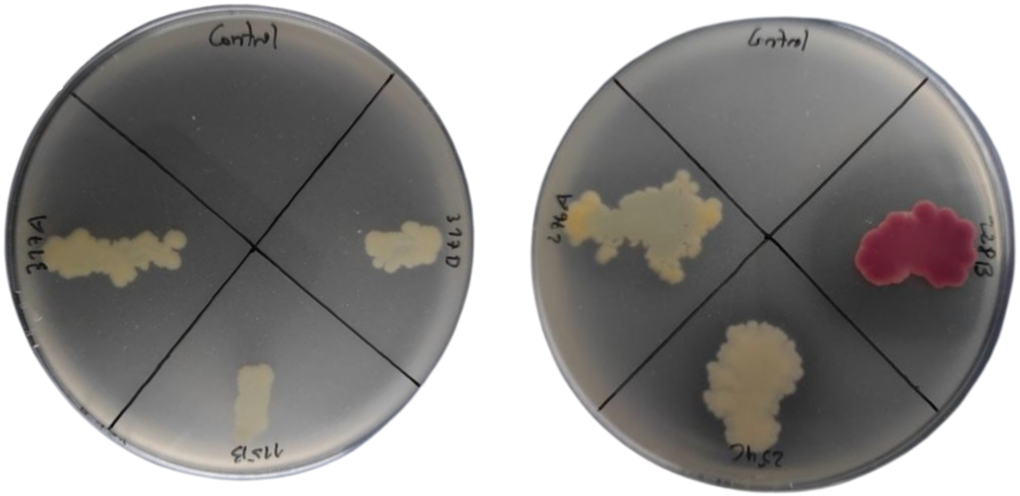

Figure S1. Microbiological screening of bacteria with gliadin-degrading activity. Randomly selected bacterial isolates obtained from stool and blood samples of CeD patients and their non-CeD first-degree relatives were cultured in TSA media supplemented with 0.2% (w/v) wheat alpha-gliadin, 37 ºC, 72 h, under aerobic conditions. (**A**) Bacteria with no gliadin-degrading activity. (**B**) Bacteria with gliadin-degrading bacteria, showing a clear halo around the bacterial colonies. Bacterial isolates with halos ≥ 5 mm were subcultured onto other gliadin plates and selected for further identification by 16S rRNA gene sequencing. Controls were inoculated with a loop with no bacterial cells.

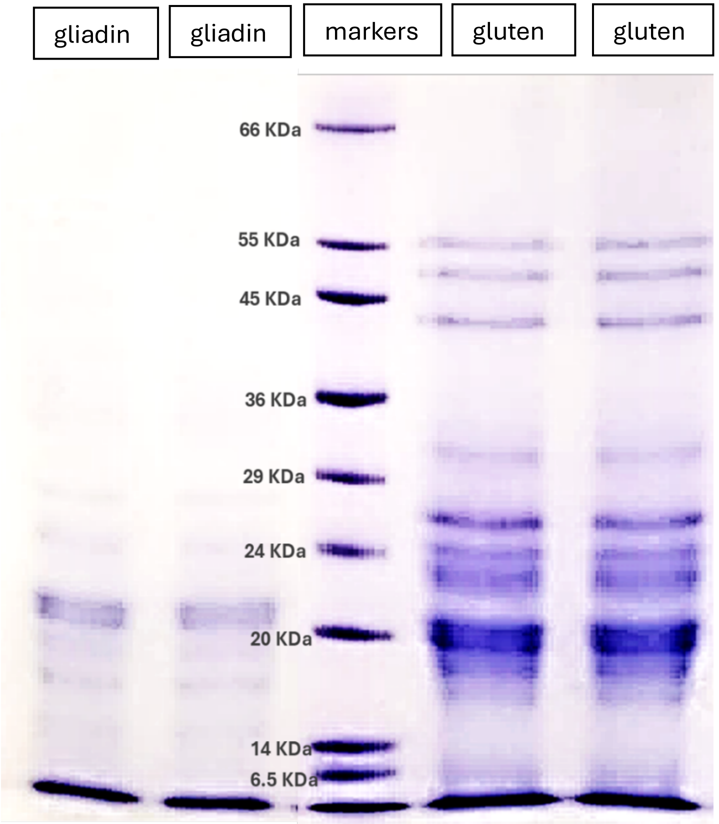

Figure S2. Coomassie Brilliant Blue stained SDS-PAGE gel of wheat (*Triticum aestivum*) gluten and gliadin. Gliadin samples were rich in alpha-gliadin (20 to 25 kDa). Gluten and gliadin samples were applied in duplicate. Protein standards (wide range markers, Sigma-Aldrich, Germany) were used for molecular weight referencing. Electrophoreses were carried out at 150 V for 1 h by making use of a Tris-glycine-SDS buffer. Gels were stained with Coomassie Brilliant Blue R-250 (Sigma-Aldrich, Germany).

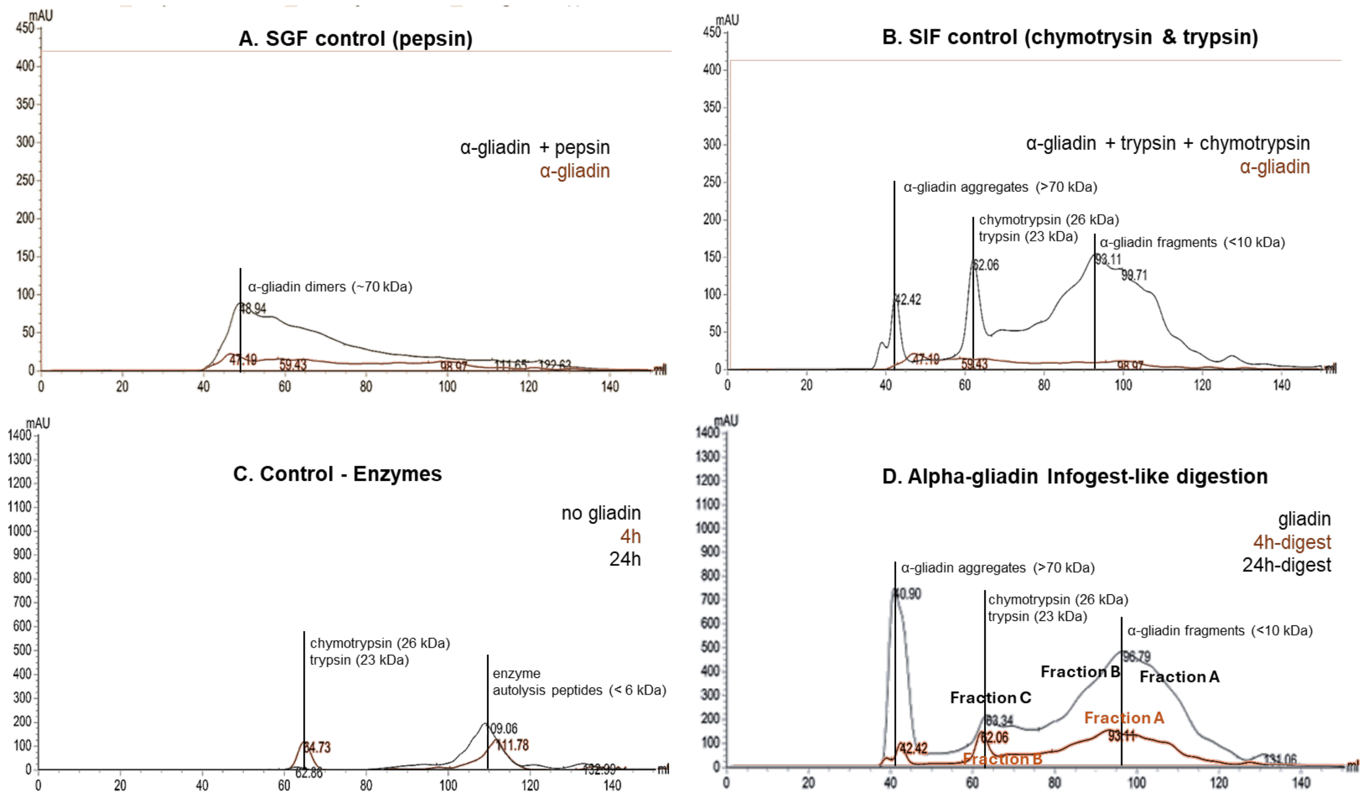

Figure S3. Size-exclusion FPLC chromatograms of enzyme controls and of the 4 h- and 24 h-digesta produced in the static in vitro INFOGEST-like digestion protocol (focused on protein digestion). (**A**) Chromatograms of undigested gliadin and pepsin-digested gliadin. (**B**) Chromatograms of undigested gliadin and (trypsin+chymotrypsin)-digested gliadin. (**C**) Chromatograms of trypsin and chymotrypsin. (**D**) Chromatograms of the 4 h- and 24 h-digesta - fractions A, B, and C were collected separately and analyzed by DLS and fluorescence spectroscopy. Apparent molecular weights were calculated by interpolation based on the elution volume versus log(molecular weight) calibration curve of protein standards.

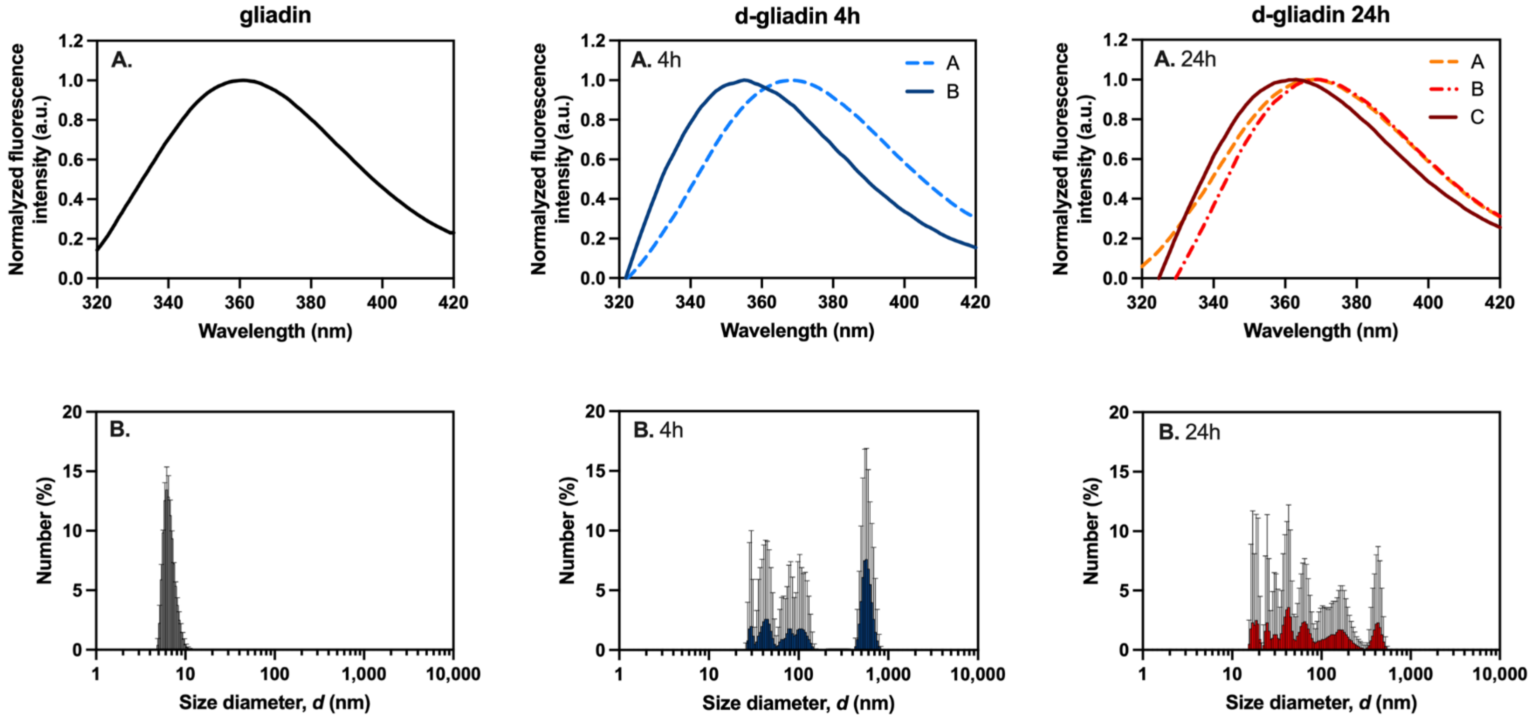

Figure S4. (**A**) Fluorescence spectra of non-digested gliadin (gliadin) and of the main FPLC fractions of digested gliadin (d-gliadin) after 4 h (A, d-gliadin peptides and B, d-gliadin intermediate aggregates – Figure S3D) and 24 h (A and B, d-gliadin peptides and C, d-gliadin intermediate aggregates, Figure S3D) of digestion, evidencing a blue-shift of the maximal emission wavelength, given the increase in d-gliadin aggregates (4h-fraction A and 24h-fraction C) with less Trp-exposure to the solvent. Spectra show normalized data, upon baseline subtraction. (**B**) DLS profiles of non-digested gliadin (gliadin) and digested gliadin (d-gliadin) exhibiting a major population of larger aggregates of d-gliadin (*diameter*, 600 nm) after 4 h of digestion and a more heterogeneous sample of d-gliadin peptides and intermediate aggregates (*diameter* < 400 nm) after 24 h.

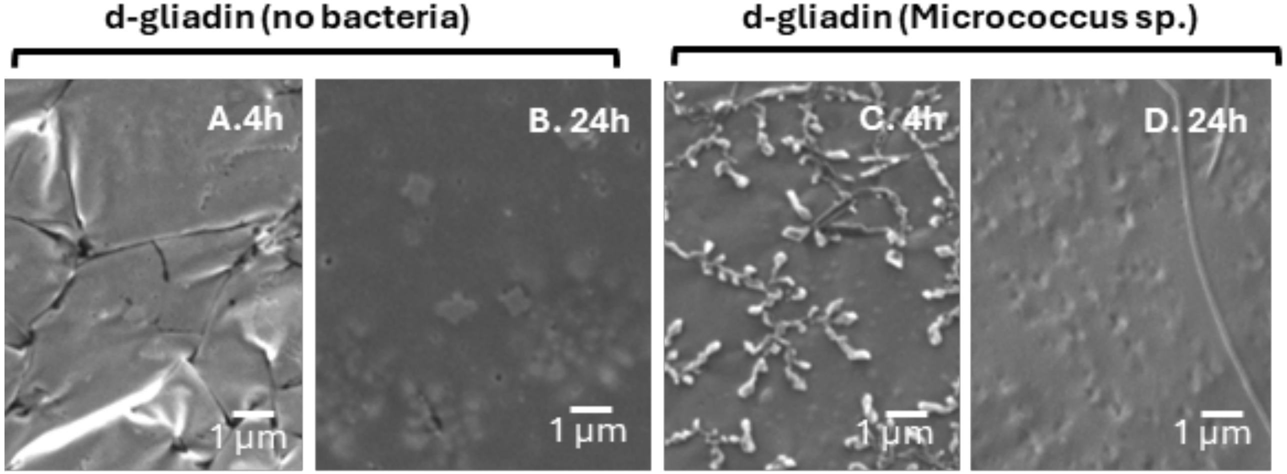

Figure S5. SEM images (3k mag.) of INFOGEST-like digested gliadin (d-gliadin) in the absence and presence of bacteria: (**A**) "Microcellular foam" of poorly digested gliadin of the 4 h-digesta without bacteria. (**B**) Smaller square-like gliadin aggregates formed after 24 h of further incubation without bacteria. (**C**) Rod-like long branched assemblies of 4 h-INFOGEST-like d-gliadin in the presence of *Micrococcus* sp. in the SIF "intestinal phase". (**D**) Small spherical aggregates formed by d-gliadin after 24 h of incubation in the presence of *Micrococcus* sp.

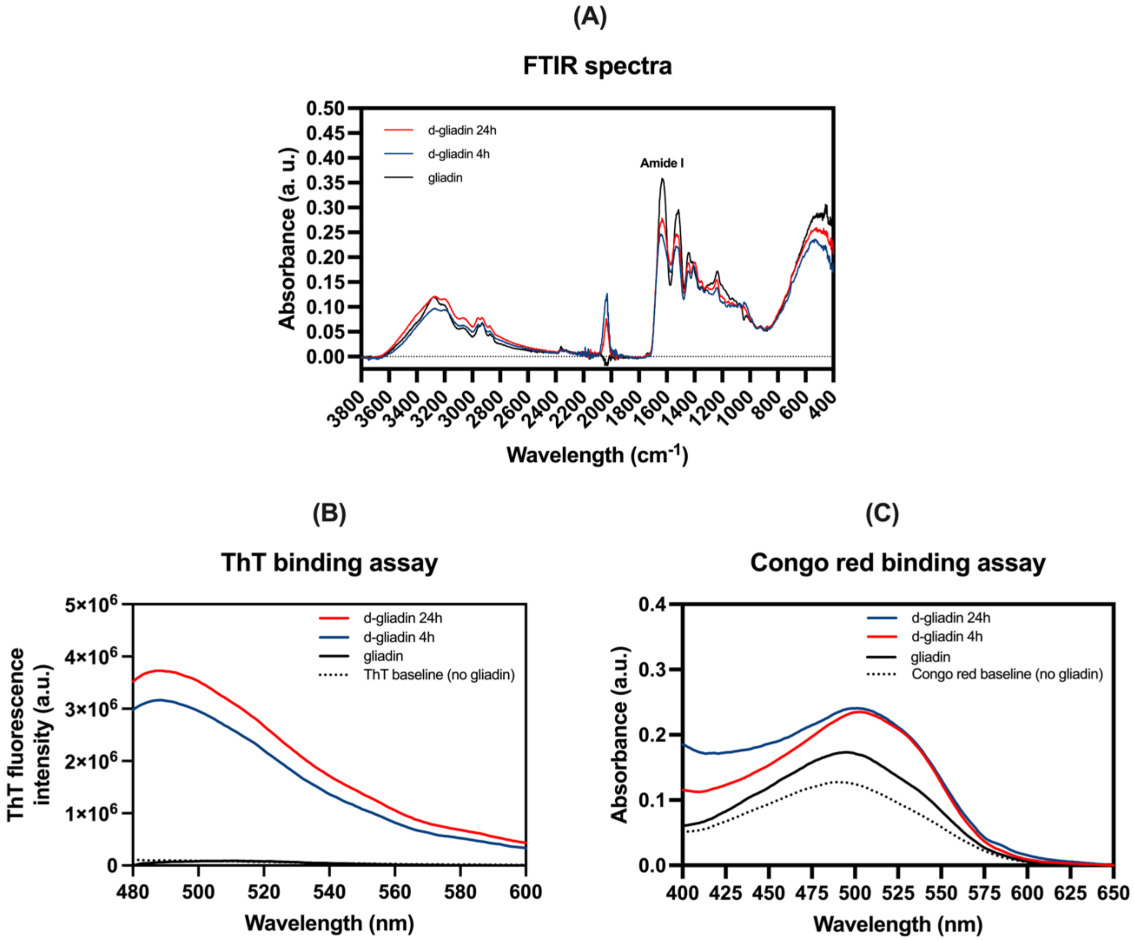

Figure S6. (**A**) Overlay of FTIR spectra of non-digested (gliadin) and digested wheat gliadin (d-gliadin) showing the typical amide A, I, II and III spectral regions found in proteins. (**B**) ThT Fluorescence emission (**C**) and Congo Red absorbance spectra reporting binding to gliadin structures (increase in ThT fluorescence and Congo Red redshift and second shoulder) after 4h of digestion and further incubation for 24h. Baseline spectra (no gliadin) of ThT and Congo red solutions in SIF, in the absence of gliadin are also shown.

4h-digests

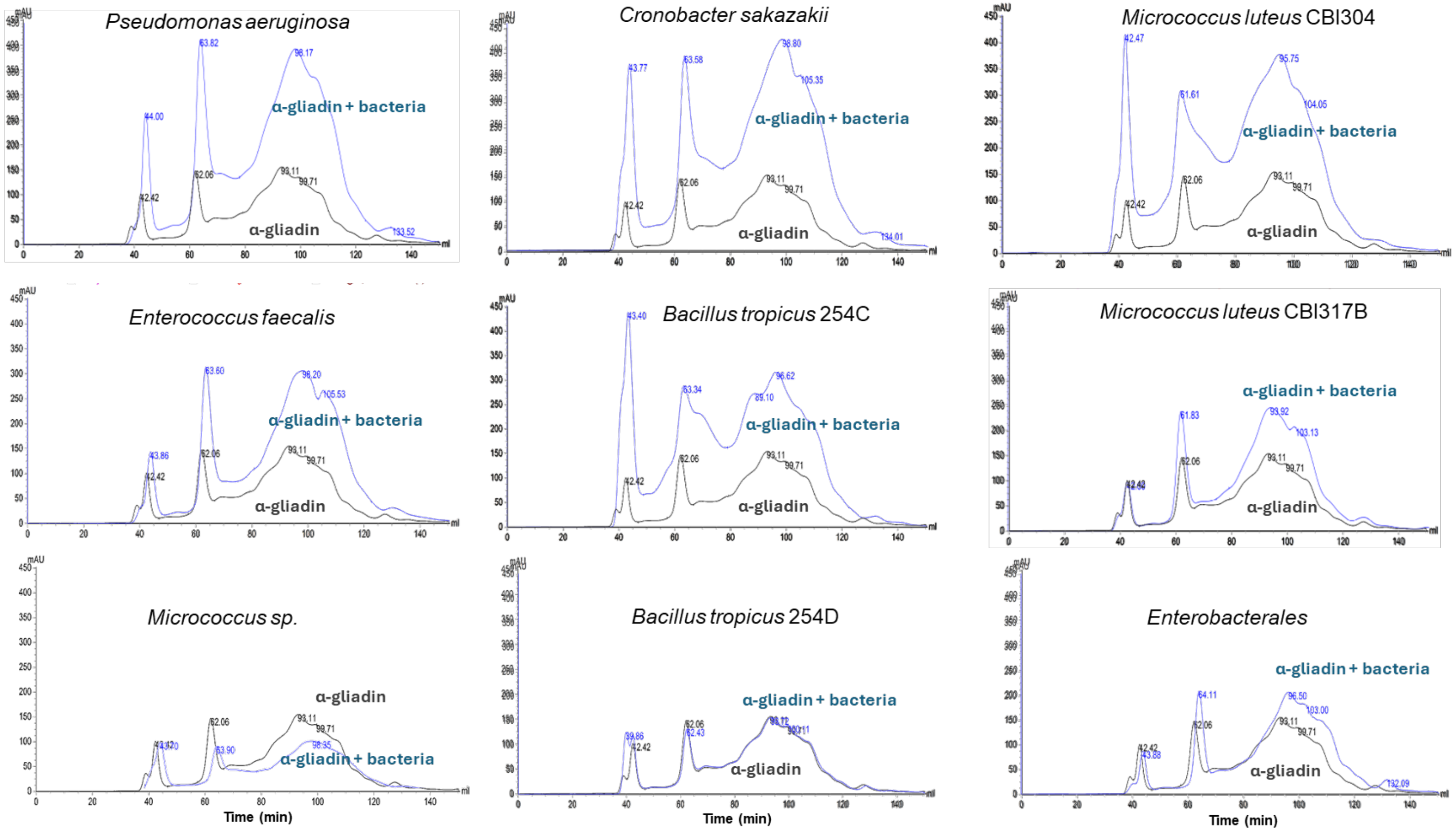

Figure S7. Size-exclusion FPLC chromatograms of gliadin 4 h-digesta (d-gliadin) in the absence (black) and presence of bacteria (blue). INFOGEST-like digestions were carried out in the presence of the various bacterial isolates in the intestinal phase (SIF): *Pseudomonas aeruginosa*, *Cronobacter sakazakii, Micrococcus luteus, Enterococcus faecalis, Bacillus tropicus*, *Micrococcus* sp., and *Serratia marcescens***.**

24h-digests

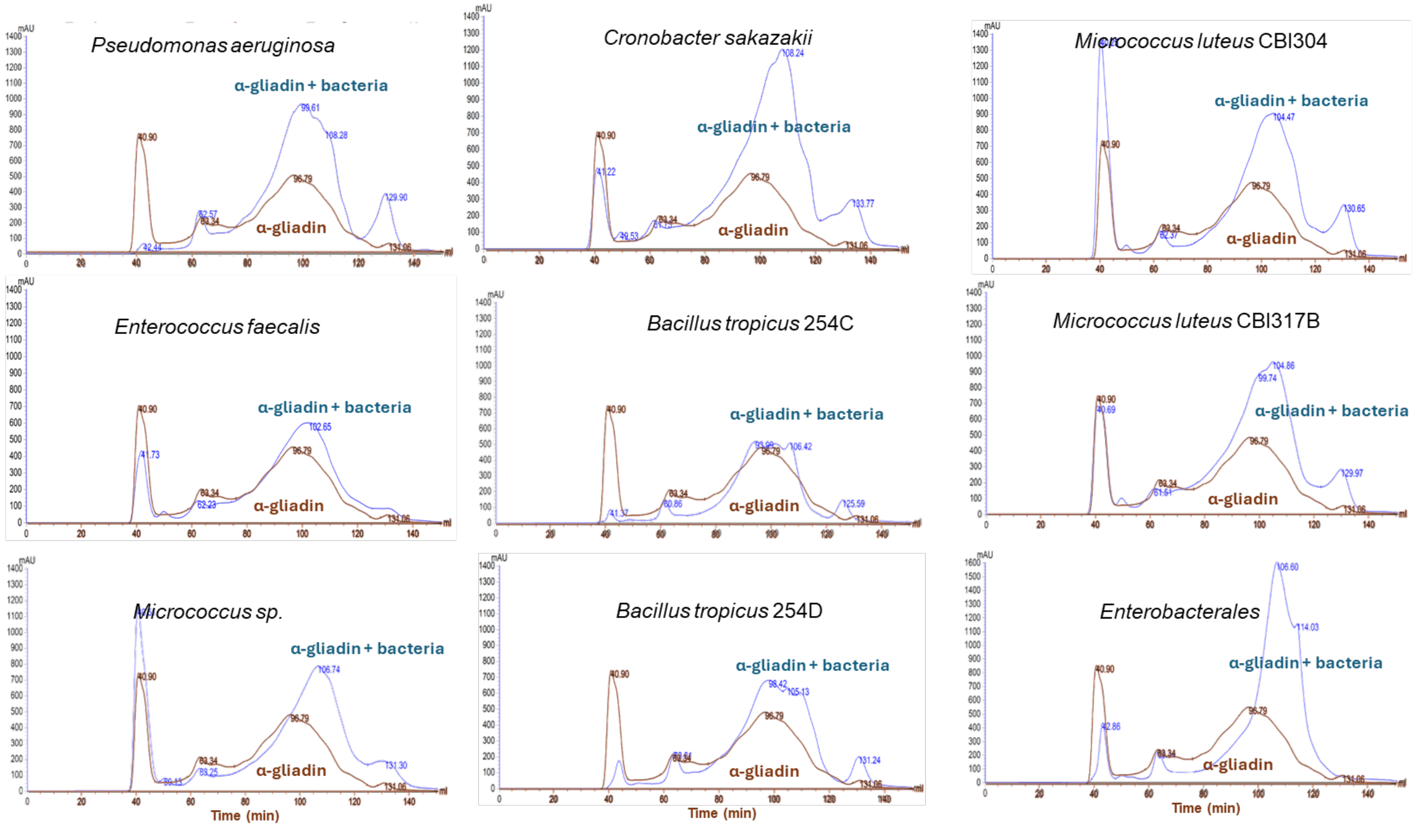

Figure S8. Size-exclusion FPLC chromatograms of gliadin after 24h of further incubation in the absence (black) and presence of bacteria (blue): *Pseudomonas aeruginosa*, *Cronobacter sakazakii*, *Micrococcus luteus*, *Enterococcus faecalis*, *Bacillus tropicus*, *Micrococcus* sp., and *Serratia marcescens*.

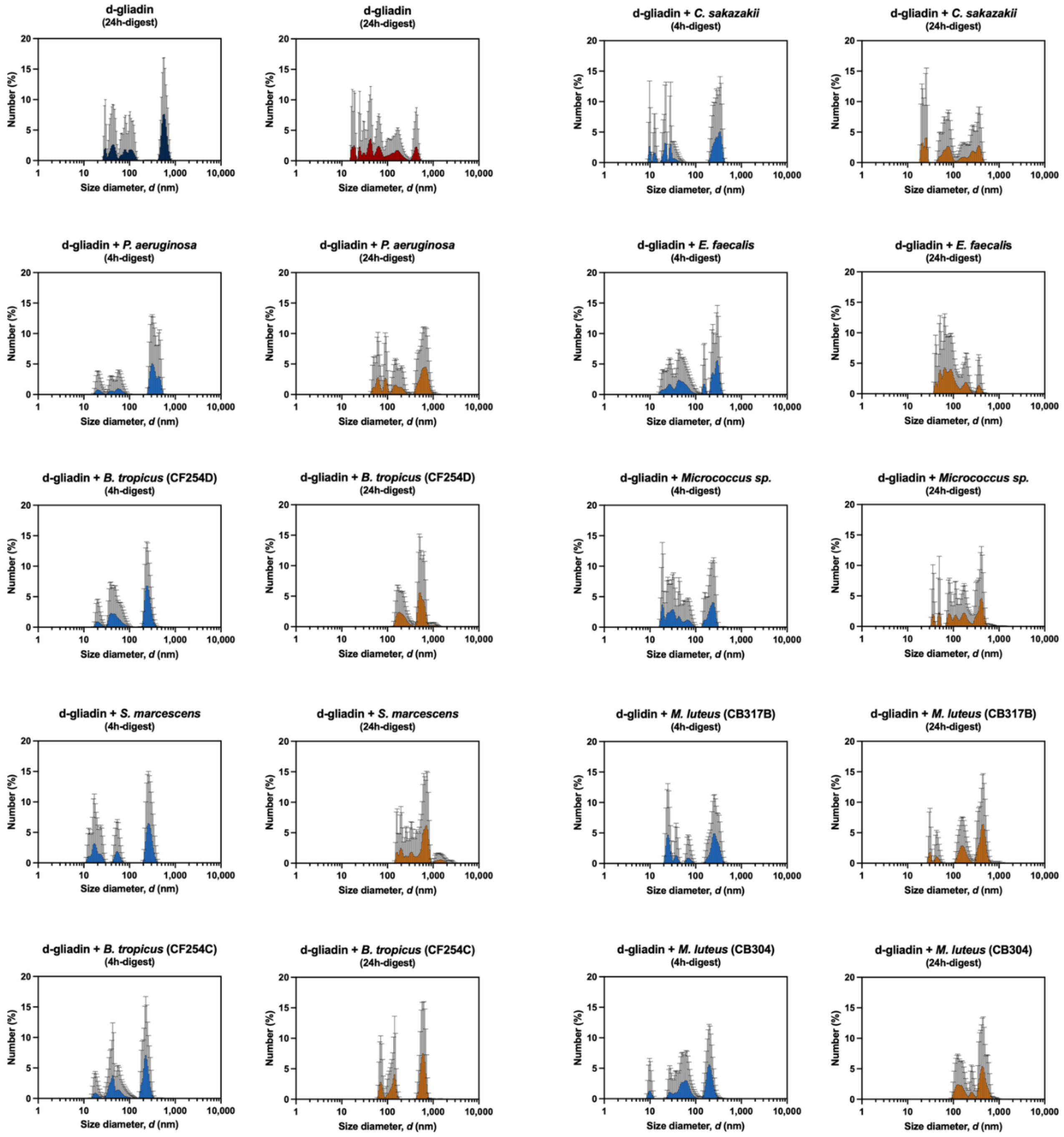

Figure S9. DLS profiles of 4 h-(blue) and 24 h-(brown) d-gliadin in the absence and presence of bacteria (*P. aeruginosa*, *S. marcescens*; *C. sakazakii*, *M. luteus*, *E. faecalis*, *B. tropicus*, *Micrococcus* sp.). After 4 h of INFOGEST-like digestion in the presence of bacteria, the DLS profiles of d-gliadin showed protein populations of smaller size (*diameter*, ~400 nm), even if after 24 h of incubation, larger particles/aggregates were also formed from d-gliadin fragment/peptide aggregation.

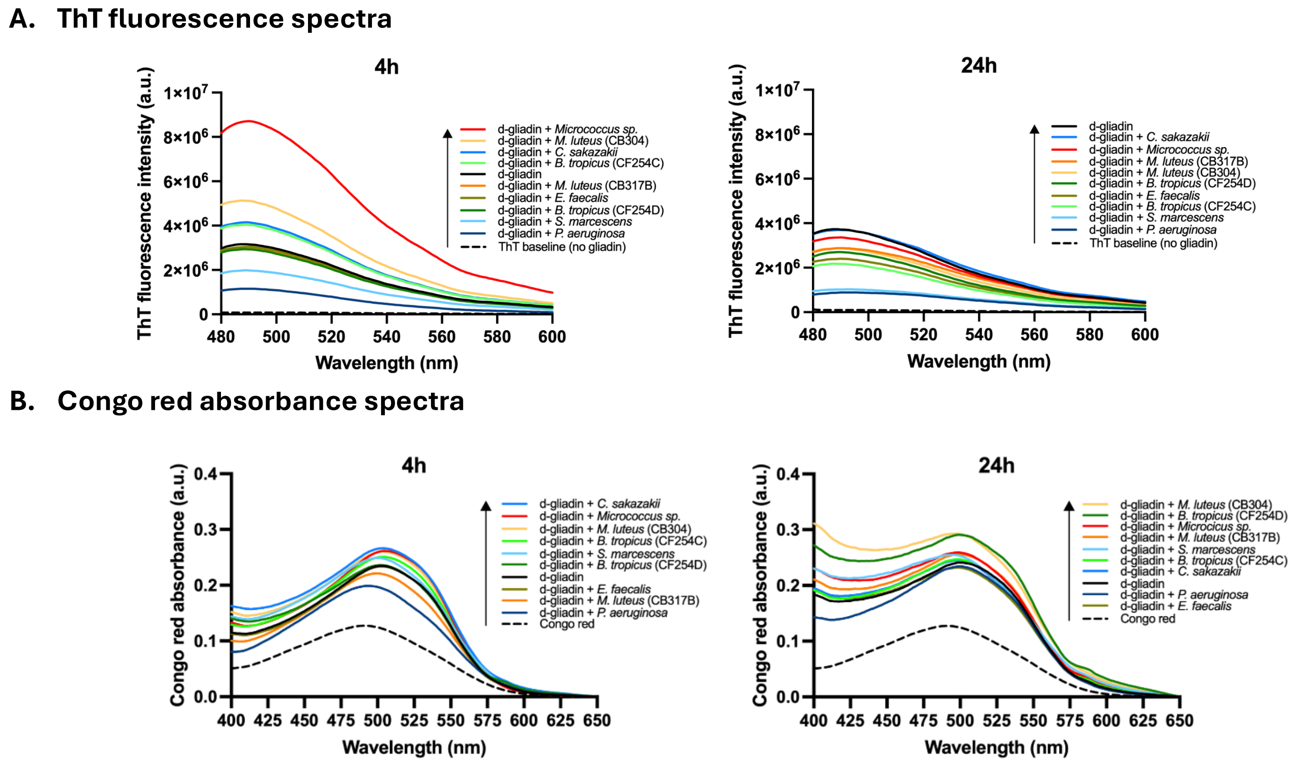

Figure S10. Characterization of the INFOGEST-like digesta of wheat gliadin (d-gliadin) produced in the presence of bacteria in the intestinal phase of the digestion. Spectra labels are listed according to increasing signal intensity. (**A**) ThT fluorescence emission and (**B**) Congo red spectra reporting differential binding of the dyes to d-gliadin. After 4 h of digestion, higher ThT fluorescence and Congo red redshifts (than d-gliadin without bacteria – solid black line) were detected for most bacterial digestions. After 24 h of incubation, all bacterial digesta presented lower ThT intensity and less Congo red redshifts than control**.**

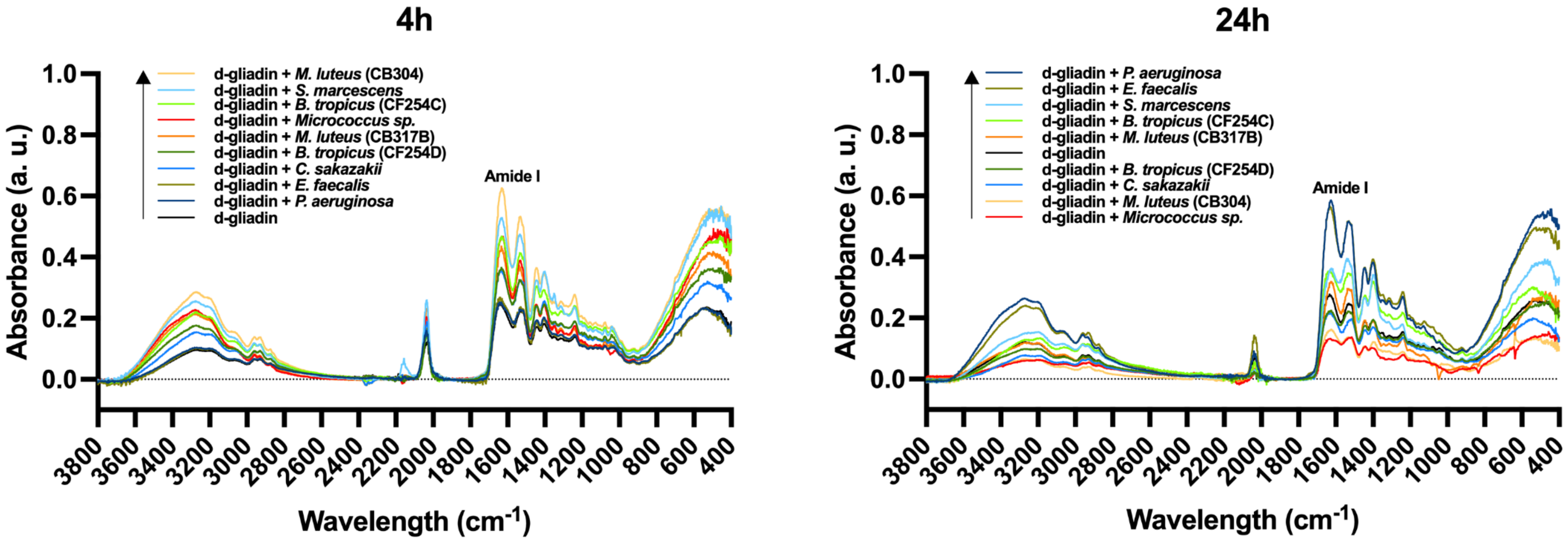

Figure S11. FTIR spectra of wheat gliadin of the INFOGEST-like digestion of digested wheat gliadin (d-gliadin) in the absence and presence of bacterial isolates (*P. aeruginosa*, *S. marcescens*, *C. sakazakii*, *M. luteus*, *E. faecalis*, *B. tropicus*, *Micrococcus* sp.) in the intestinal phase SIF of the digestion process, after 4 h (left) and 24 h (right) of incubation.
